# Capturing Current Practices and Characterizing Measurement Reproducibility for Seized Drug Analysis Using Ambient Ionization Mass Spectrometry: An Interlaboratory Study

**DOI:** 10.1021/jasms.5c00213

**Published:** 2025-08-27

**Authors:** Edward Sisco, Dennis D. Leber, Arun S. Moorthy

**Affiliations:** † 10833 National Institute of Standards and Technology, Gaithersburg, Maryland 20899, United States; ‡ Department of Forensic Science, 6515 Trent University, Peterborough, Ontario K9L 0G2, Canada

**Keywords:** Ambient Ionization Mass Spectrometry, DART-MS, ASAP-MS, Seized Drugs, Spectral Reproducibility, Interlaboratory Study

## Abstract

The use of ambient ionization mass spectrometry (AI-MS) to aid in the preliminary screening of seized drug evidence has steadily increased over the past two decades. Unlike gas chromatography–mass spectrometry (GC-MS), where electron ionization using a single quadrupole analyzer is commonplace, a wide range of ionization sources and mass spectrometers can be used in AI-MS. Differences in instrument configuration can lead to substantial variability in the mass spectral data obtained. An interlaboratory study, consisting of 35 participants from 17 laboratories, was conducted to begin to understand the landscape and the differences in the data that are produced. Laboratories analyzed a series of 21 solutions across multiple days using their own instrumental methods. Mass spectra were extracted and compared to understand operator, within-lab, and between-lab reproducibility for common compounds and mixtures observed in seized drug analysis. In addition, five participants analyzed the 21 solutions using prescribed method parameters to measure reproducibility improvements when using identical instrumental conditions. Mass spectral reproducibility, measured through pairwise cosine similarity, was found to be generally quite high, regardless of sample type, instrument type, method, or operator. Low-fragmentation spectra showed the lowest variability, as they were dominated by intact protonated molecule peaks. Several potential issues that increased variability were identified, including carryover from mass calibrants, poor sample introduction, and mass spectrometer inlets that required cleaning. The use of uniform method parameters was shown to increase the reproducibility of mass spectra across laboratories, most notably at higher in-source collision-induced dissociation energies. This study provides initial insights into the current landscape of AI-MS in seized drug analysis and lays the foundation for future studies that can provide needed data for the development of documentary standards, standard methods, and possibly the establishment of error rates.

## Graphical abstract

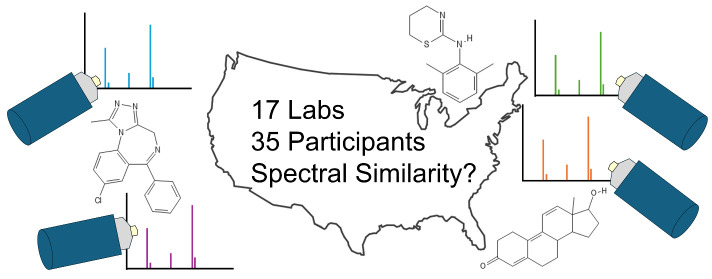

## 1 Introduction

Seized drug analysis remains one of the most frequently requested types of forensic analyses in the United States, with over 1 million reports issued in 2022.^1^ Over the past decade, the job of the forensic drug chemist has seen added challenges due to increasing numbers of submissions, more complex samples, and a dynamic drug supply. To address these concerns, laboratories are increasingly seeking to implement new analytical tools that can provide rapid, specific, and sensitive data as early in the workflow as possible. Ambient ionization mass spectrometry (AI-MS) is one of the analytical tools that can address these needs.

AI-MS tools have a significant history in seized drug analysis, dating back to initial demonstrations of direct analysis in real-time mass spectrometry (DART-MS) for drug analysis by Cody et al. in 2005 ^2^ and the first validation of a method for forensic drug analysis by Steiner and Larson in 2009.^3^ Since then, implementation of DART-MS into practicing forensic laboratories has increased. Research surrounding how to best implement this approach for a variety of different sample types has also been demonstrated.^4,5^ In addition to the growing research in this field, the availability of resources such as validation templates,^6^ spectral libraries,^7^ and search software^8^ has further lowered the barriers to implementation.

Other AI-MS approaches have also been researched and either are currently in use or hold potential for application for illicit drug analysis. Atmospheric solid analysis probe (ASAP) has been demonstrated to effectively analyze drug seizures ranging from powders and pills^9^ to more unique sample matrices.^10^ This approach has also been adopted by forensic laboratories and may be appealing due to the lack of helium required for ionization. Paper spray ionization (PSI),^11^ direct sample analysis (DSA),^12^ desorption electrospray ionization (DESI),^13^ and dielectric barrier discharge ionization (DBDI)^14^ have also been demonstrated for the analysis of seized drug samples, although their adoption by practicing forensic laboratories remains limited.

While the adoption of AI-MS into the seized drug laboratory can help address pain points in the analysis chain, by offering rapid, comprehensive information with minimal sample pretreatment, it is not without its challenges and limitations. The wide range of AI sources and mass spectrometer types means that laboratories implementing AI-MS could use vastly different systems. Source, mass spectrometer, and instrumental method differences could generate significant data variations, driving differences in fragmentation, specificity, and sensitivity. Beyond the instrument itself, sample introduction for many of these techniques is manual. Manual sample introduction can further increase variation in mass spectra within a laboratory, where multiple analysts use the same instrument. AI sources, as the name implies, ionize the sample under ambient conditions, meaning that changes in the environment can influence how effectively analytes are ionized. For instance, previous studies have shown that changes in humidity,^15,16^ the presence of solvent vapors,^17^ and environmental contaminants can alter spectral data obtained from a sample.

While mass spectrometry is a well-established technique capable of producing highly reproducible data across laboratories when using standardized ionization methods and parameters such as 70 eV electron ionization, the implementation of AI increases the likelihood that variability at the intra- and interlaboratory level will be greater than in traditional approaches. Attempts to understand the effects of mass spectral variability in AI-MS systems have been somewhat limited in the general literature and almost entirely unexplored within a forensic context. A pair of interlaboratory studies conducted in 2015 and 2017 looked at possible challenges for qualitative and quantitative analyses using AI-MS.^18^ The data from the first study highlighted commonalities and differences in the ability of an AI-MS user to detect a wide range of compounds, showing that low polarity and negative ion compounds posed the greatest challenges. Visual differences in spectra produced by using the same ionization source and mass spectrometer at different laboratories were also observed. Quantitation was possible; however, the need for an internal standard to address variability issues was noted. Obtaining a reproducible signal from samples in complex matrices was also a challenge.^18^ Another interlaboratory study looking at the feasibility of using an existing spectral database for wood identification across multiple platforms was conducted.^19^ To our knowledge, the literature focusing on understanding AI-MS reproducibility beyond these studies has been largely focused on either understanding similarities among spectra from different ionization sources on the same mass spectrometer or comparing spectra to spectral libraries generated on different mass spectrometers.^20^

In this work, we discuss the results from an AI-MS interlaboratory study that focused on understanding mass spectral variability within the context of seized drug analysis. The study involved 35 operators from 17 laboratories. Each operator made replicate measurements of a set of 21 solutions during four discrete measurement sessions. Mass spectral data were combined with method information and other metadata to address three questions:

(1) What methods and instrumentation are used in seized drug laboratories in the United States?
(2) What is the operator, intralaboratory, and interlaboratory mass spectral variability for compounds and mixtures of relevance to seized drug analysis, and what factors drive variability differences?
(3) Does the use of predefined method parameters decrease interlaboratory mass spectral variability?

## 2 Materials and Methods

### 2.1 Interlaboratory Study Design and Data Collection

The interlaboratory study was conducted in the summer of 2023, and data analysis took place throughout 2024. Requirements to participate in the study included (1) being an accredited forensic laboratory or research laboratory in the United States with a valid Schedule I and II DEA license, (2) having an AI-MS system and trained operators, and ((3) being willing to complete the entire study. Laboratories were encouraged to have multiple operators participate in the study.

The interlaboratory study consisted of (1) a prestudy survey to capture information about the operator, laboratory, instrument, and method(s), (2) four independent measurement sessions where an operator analyzed a study kit containing 21 solutions using a method of their choosing, (3) an optional component identical to (2) where method parameters were prescribed, and (4) a poststudy survey. All operators were required to complete parts 1, 2, and 4. Optional component 3 was limited to laboratories with a JEOL AccuTOF (Peabody, MA, USA) mass spectrometer.

All laboratories were provided with a study kit that contained 21 solutions, a thermometer (Switchbot Thermometer and Hygrometer Plus, Switchbot Inc., Wilmington, DE, USA), prelabeled vials (2 mL amber glass vials, Restek, Bellefonte, PA, USA) to transfer ampuled solutions into, and sheets for recording sample introduction times. For laboratories that had one or two operators, one solution kit was provided. For laboratories that had three or more operators, multiple solution kits were provided. The solution set contained 16 single-compound solutions and five multi-compound solutions that were created, ampuled, packaged, and shipped by Cayman Chemical (Ann Arbor, MI, USA). Solution contents were verified by NIST prior to the sets being shipped to participants. Since this was not a blind study, the identity of the solutions (Table 1) was provided to the operators.

**Table 1:** Solutions Included in the Kits Provided to Participating Laboratories^a^

**Solution #**	**Contents**		**Solution #**	**Contents**
1	Acetyl fentanyl·HCl		17 (Mix 1)	Cocaine·HCl (0.7 mg/mL)
2	Alprazolam			Levamisole·HCl (0.3 mg/mL)
3	Amphetamine·HCl		18 (Mix 2)	Caffeine (0.25 mg/mL)
4	Benzyl fentanyl·HCl			Fentanyl·HCl (0.05 mg/mL)
5	Caffeine			Heroin (0.2 mg/mL)
6	Cocaine			Xylazine·HCl (0.5 mg/mL)
7	Fentanyl·HCl		19 (Mix 3)	Methamphetamine·HCl (0.7 mg/mL)
8 (ACN)	Heroin			Phenylephrine (0.3 mg/mL)
9	Levamisole·HCl		20 (Mix 4)	Alprazolam (0.2 mg/mL)
10	Methamphetamine·HCl			Fentanyl·HCl (0.1 mg/mL)
11	Phentermine·HCl			Heroin (0.4 mg/mL)
12	Phenylephrine			Quinine (0.3 mg/mL)
13	Quinine		21 (Mix 5)	Acetyl fentanyl·HCl (0.33 mg/mL)
14	Tramadol·HCl			Benzyl fentanyl·HCl (0.33 mg/mL)
15	Trenbolone			Methamphetamine·HCl (0.33 mg/mL)
16	Xylazine·HCl			

a Single-component solutions had a nominal concentration of 0.25 mg/mL. For multi-component solutions, the concentration of each compound is provided in the table. Solutions were prepared in methanol unless noted as acetonitrile (ACN).

Operators were asked to analyze each of the 21 solutions in triplicate, along with any positive or negative controls they would employ in routine analysis. This was defined as a *measurement session*. Operators were asked to complete a total of four measurement sessions across multiple days and times of the day (two morning sessions and two afternoon sessions, with no more than two sessions completed on a given day). For each measurement session, operators were asked to record the ambient temperature and humidity of the laboratory—using the provided thermometer/hydrometer—and to fill out a run sheet which denoted the approximate times the positive control, negative control, and solution were analyzed within a run. Operators were asked to follow their standard operating procedures, if any, regarding mass calibration and/or mass drift compensation processing before sending the datafiles and run sheets back to the study organizers. A predefined file naming convention was used to simplify data processing. The NIST Research Protections Office reviewed the study protocol and determined this interlaboratory study was not human subjects research.

### 2.2 Data Conversion and Mass Spectra Extraction

Once received, all datafiles were converted to an open format—either to network Common Data Form (.netCDF) or mass spectrometry specific extensible markup language (.mzXML), depending on the available conversion tools. Table 2 provides a list of the software tools used for data conversion, as well as the original file formats and the formats into which they were converted. The run sheets, which were received as scanned portable document format (.PDF) files, were manually converted into spreadsheets to aid in automated mass spectral extraction.

**Table 2:** Datafile Formats Received, Data Conversion Tools Used, and Open-Source File Formats to Which Data Were Converted

**Received Data Format**	**Conversion Software Used**	**Open-Source Format of Converted Data**
JEOL msAxel Datafile	msAxel Data Converter	.netCDF
JEOL MassCenter Datafile (.7rw)	MassCenter Data Converter	.netCDF
Thermo (.raw)	Proteo Wizard MSConvert Tool	.mzXML
Waters (.raw)	Proteo Wizard MSConvert Tool	.mzXML

For each data file, an *aggregated mass spectrum* was generated using a custom R-script. The R-script requires (1) the datafile in one of two open formats (.mzXML or. netCDF), (2) the sample introduction start and stop times in minutes from the run sheet, (3) the known protonated molecular mass of the sample being measured, (4) the mass accuracy of the mass spectrometer being used to measure the sample (obtained in the presurvey), (5) a threshold value that determines the minimum relative ion current for a raw mass spectrum to be considered appropriate for inclusion in the aggregated sample mass spectrum, and (6) a threshold value that determines the minimum relative intensity of a peak to be stored in the aggregated mass spectrum. For this study, a relative ion current threshold of 20% and a relative peak intensity threshold of 1% were used. Figure 1 illustrates key steps of the aggregation process. In Figure 1a, we present an example data file (chronogram) with red lines indicating the known start and end points of when the sample was introduced to the instrument, the green line indicating the ion current threshold used to determine that there was sufficient signal to produce relevant mass spectra, and the blue points indicating the time locations of the mass spectra that were used to generate the aggregated mass spectrum. The aggregated mass spectra were generated by summing all individual mass spectra and then normalizing them to the highest intensity peak.

**Figure 1:**
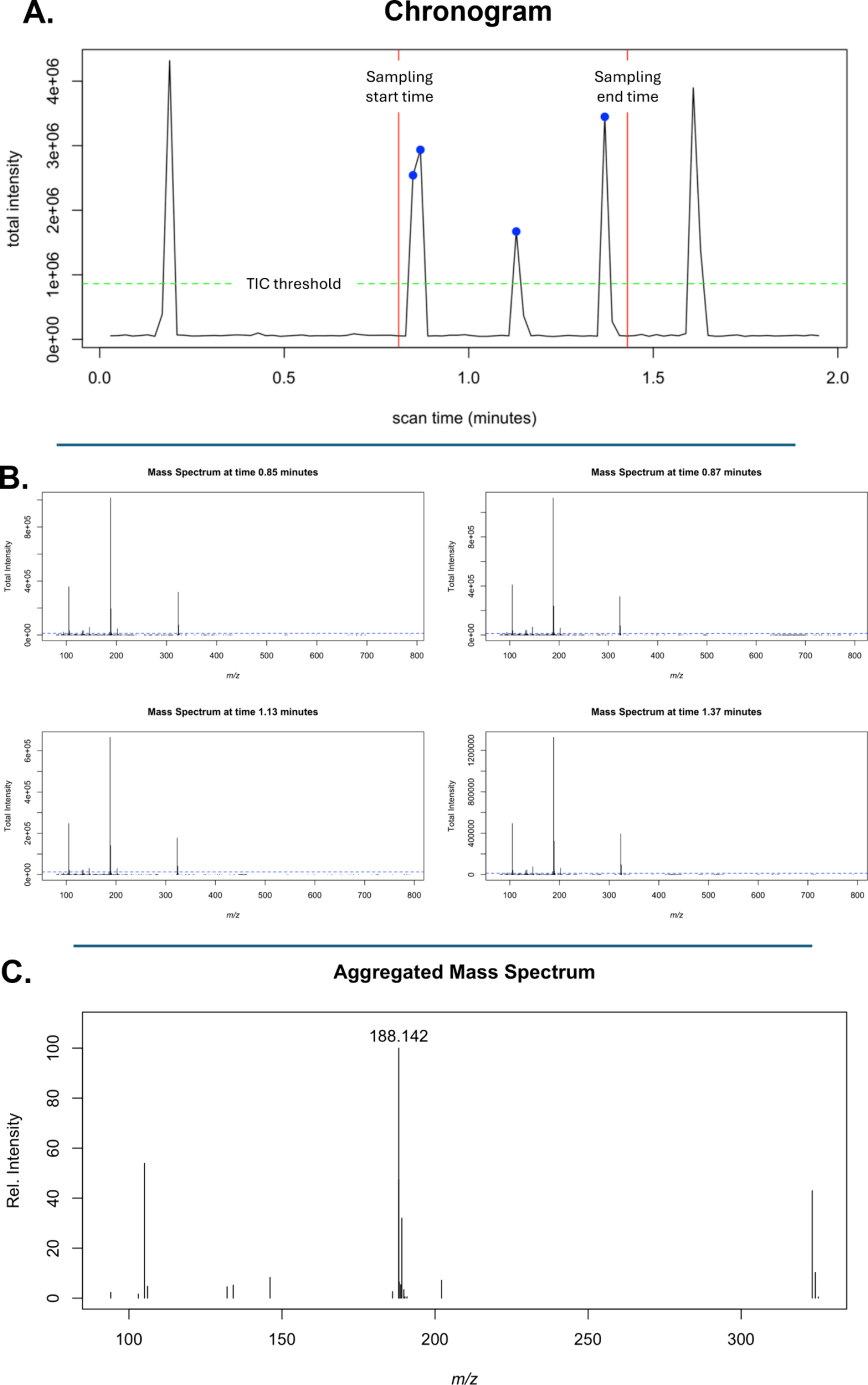
Steps to generate an aggregated mass spectrum from a raw data file. (A) TIC threshold and runtime information are used to identify raw mass spectra of sample, with time locations indicated by blue points. (B) Identified mass spectra are pulled, and peaks less than a spectral threshold (shown in dotted blue line) are removed. (C) All spectra are merged by binning with a grid as determined by the sample’s known protonated molecular mass and the mass tolerance of the instrument used to measure the sample.

For laboratories that provided data at one *is-*CID energy, spectra were defined as low-fragmentation energy. For laboratories that provided data at three *is*-CID energies, spectra were defined as low-, mid-, and high-fragmentation energy based on increasing *is*-CID voltage. For laboratories that provided data at four *is-*CID energies, the lowest *is-*CID voltage data was ignored and the remaining three were defined as low-, mid-, and high-fragmentation energy, based on increasing *is*-CID voltage. The lowest *is-*CID voltage data was dropped in these instances to allow us to align voltages with laboratories using JEOL AccuTOF mass spectrometers that collected three *is*-CID voltages.

### 2.3 Characterizing Measurement Reproducibility

Given a set of mass spectra for a specified sample, we define the *reproducibility index* of a set of mass spectra as the *minimum pairwise similarity* computed between all possible unique pairs of mass spectra in the set. We computed pairwise mass spectral similarity using cosine similarity as described in ref 21, and so the computed reproducibility indices take values between 0 a.u. and 1 a.u., inclusive. Larger values (approaching 1 a.u.) imply that the spectra in the set are similar and thus reproducible measurements. To enable comparison of nominal mass and high-resolution spectra, all high-resolution spectra were binned prior to any comparison being conducted.

When the set of mass spectra being compared was measured at the same lab, we refer to the computed reproducibility index as the *Lab Reproducibility Index (LRI)*. When the set of mass spectra being compared was measured by a single operator, we refer to the reproducibility index as the *Operator Reproducibility Index (ORI)*.

## 3 Results and Discussion

### 3.1 Current AI-MS Landscape

In total, 35 individuals from 17 laboratories participated in the study. More than half (*n* = 9) of the laboratories had a single individual participate, although up to five individuals from the same laboratory participated. The 17 laboratories were dispersed among state and local forensic laboratories (*n* = 8), federal forensic laboratories (*n* = 8), and federal research laboratories (*n* = 1). Experience in using AI-MS varied among the individuals who completed the study; the slight majority (*n* = 12, 35%) had less than one year of experience using AI-MS, 32% (*n* = 11) had between one and five years of experience, and 32% (*n* = 11) had greater than five years of experience. One operator did not provide information on experience.

Five (29%) of the laboratory buildings were greater than 20 years old, 11 (65%) were between five and 20 years old, and one (6%) was less than five years old. One lab did not provide information about building age. Operators were also asked whether a fume extractor, which can be used to prevent exposure of toxic fumes, was used with the AI-MS setup. Nine (53%) laboratories reported using a fume extractor, six (35%) reported not using a fume extractor, and two (11%) laboratories did not provide an answer.

Direct analysis in real time (DART, Bruker Daltonics, Billerica, MA, USA) was the ionization source used by all of the laboratories except for one that used an atmospheric solid analysis probe (ASAP) source (Waters Corporation, Milford, MA, USA). Mass spectrometers used included the JEOL AccuTOF (*n* = 13, 72%) (JEOL USA, Peabody, MA, USA), Thermo Fortis Plus (*n* = 2, 11%) (Thermo, Waltham, MA, USA), Thermo Q-Exactive (*n* = 1, 6%), and Waters QDa/RADIAN (*n* = 2, 11%). One laboratory used two types of mass spectrometers. The age of instrumentation varied, with three (17%) being less than two years old, eight (44%) being between two and five years old, and six (33%) being more than five years old. One laboratory did not provide information on instrument age.

Although various sampling methods can be used with DART-MS, all but one laboratory reported using manual sampling, either glass microcapillaries (*n* = 15, 94%) or PTFE swabs (*n* = 1, 6%). The laboratory not conducting manual sample introduction used a linear rail configuration with DipIt tips for automated sample analysis.

Laboratories that completed mass calibration used either poly­(ethylene glycol) (for those using a JEOL AccuTOF) or Piece EMRS solution (for those using a Thermo Fortis Plus). For positive controls, laboratories employed a range of compounds: AB-FUBINACA, acetaminophen, caffeine, cocaine, dextromethorphan, diltiazem, linoleic acid, mannitol, methamphetamine, nefazodone, sildenafil, or mixtures of these compounds. Eight laboratories reported using a negative control, which consisted of either a solvent blank or a sampling media blank (i.e., clean microcapillary rod or PTFE wipe). One laboratory reported that they do not run a negative control.

All laboratories operated their AI-MS system in positive ionization mode. All but one that used DART used it with helium as the source gas; one laboratory used nitrogen. DART source gas temperatures ranged from 250 to 400 °C, and exit grid voltages ranged from 50 to 350 V. All laboratories used the 2.5 mm ID ceramic cap, except the laboratory that employed the linear rail, which used the 0.5 mm ID cap. All laboratories employing DART used the DART-SVP version of the ionization source, aside from one laboratory, which used the older DART-100 version. More detailed information on laboratory parameters can be found in Table S1.

As expected, the mass spectrometer parameters varied significantly and depended greatly on the type of mass spectrometer being used. Orifice temperatures ranged from 80 to 450 °C. Mass scan ranges spanned *m*/*z* 10 (lowest *m*/*z*) to *m*/*z* 1250 (highest *m*/*z*), though all laboratories collected data within the *m*/*z* 80 to *m*/*z* 600 range. Two (11%) laboratories used MS/MS collection modes, four (21%) laboratories collected full-scan data, and 13 (68%) laboratories collected multiple full-scan spectra at differing in-source collision-induced dissociation (*is*-CID) energies (two laboratories had operators who collected different types of data). Product ion scan (MS/MS) data was not investigated due to the limited number of laboratories that provided it.

### 3.2 Mass Spectral Reproducibility

#### 3.2.1 Overall Mass Spectral Reproducibility

An initial investigation of overall mass spectral reproducibility was conducted visually by generating heat maps of all mass spectra collected by all operators for a given compound and energy level. The heat maps, examples of which are provided in Figure 2, enabled identification of two possible issues that affect reproducibility at the lab or operator level. The first issue is shown in the acetyl fentanyl spectra (Figure 2A–C) generated by Operator 1-1. In these spectra, a signature reminiscent of a polymer can be seen in the data along with the protonated molecule for acetyl fentanyl. There are two possible reasons for this observation: either there is carryover of the polyethylene glycol (PEG) that was used for calibration of the mass spectrometer (more likely) or there is contamination on the glass microcapillary rod used for sampling. The glass rods that are commonly employed in DART-MS analysis are shipped in plastic packaging that generates a noticeable signature of plasticizers on the rod if not properly baked off before analysis. The mass spectra generated by Operator 1-1 for Mix 2 (Figure 2D–F) did not show the same polymer distribution, further lending credence to carryover from PEG used for mass calibration.

**Figure 2:**
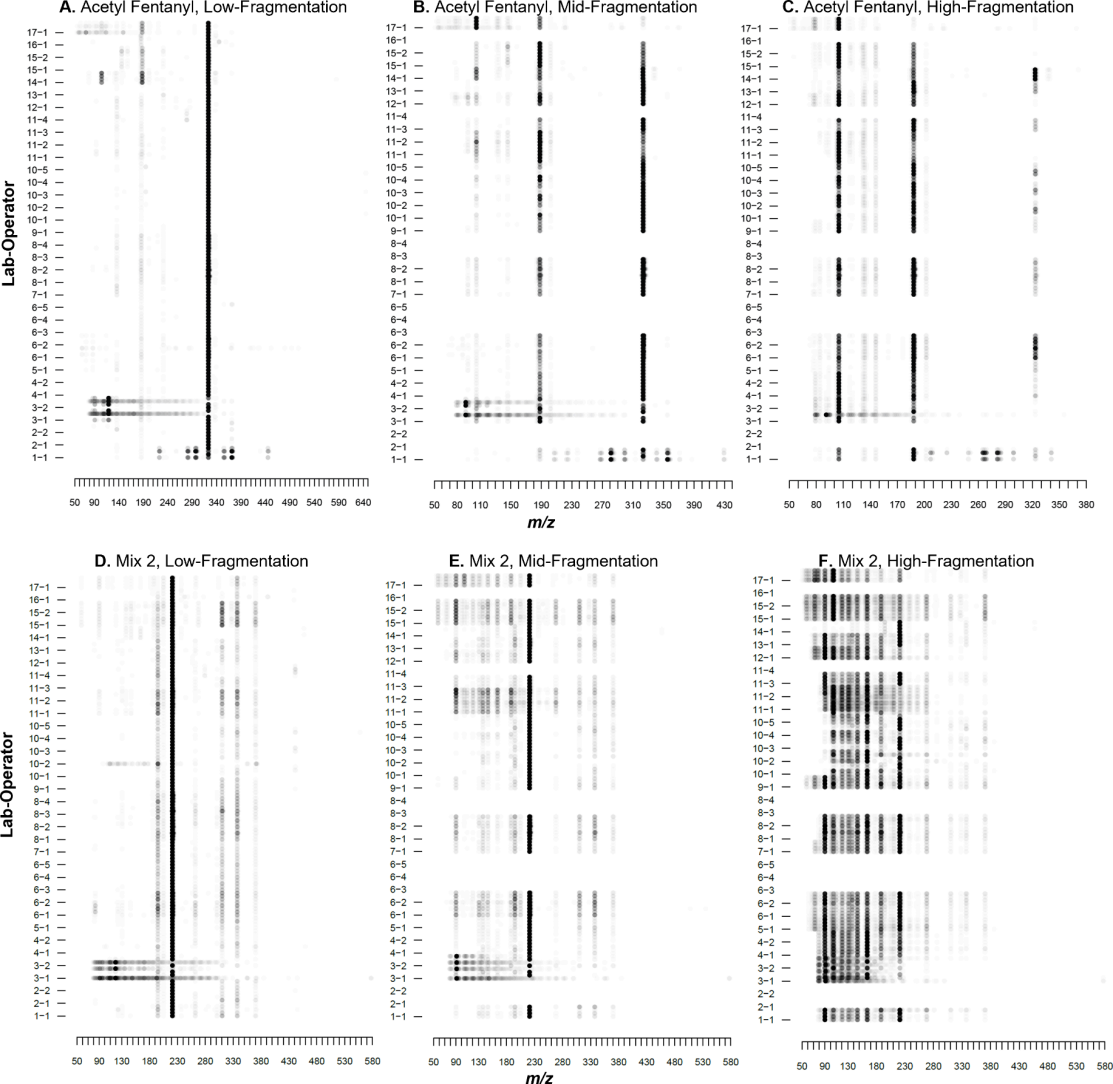
Example heat maps using aggregated *is*-CID mass spectral measurements of (A–C) acetyl fentanyl and (D–F) Mix 2 from operators at (A,D) low-fragmentation energy, (B,E) mid-fragmentation energy, and (C,F) high-fragmentation energy. Each operator completed four measurement sessions, resulting in four aggregated spectra per operator. Note that not all operators provided measurements at the mid- and high-fragmentation levels. Heat maps for all compounds and mixtures analyzed in this study are provided in Figures S2–S20.

The second issue is poor sample introduction, resulting in noisy mass spectra, as seen with Operators 3-1 and 3-2 (Figure 2). Spectra produced by both operators are visually irreproducible, with some replicates aligning with the general trend, while others show significant high-intensity peaks in the sub-*m*/*z* 150 region. The chronographic data for these operators were further investigated to determine what may be driving the irreproducibility. The likely reason for the noisy data generated was attributed to insufficient time in the DART gas stream for the sample to be thermally desorbed. Chronograms and extracted ion chronograms (EICs) for the protonated molecules for peaks of interest showed multiple instances where no significant chronographic peak was observed above background levels and the EIC signal barely rose above the baseline (Figure S1), indicating that either the sample was not left in the DART gas stream long enough to thermally desorb or the operator did not intersect the DART gas stream to enable efficient thermal desorption.

Interestingly, the large majority of spectra obtained were visually similar regardless of the analytical method, AI-MS system, and fragmentation level used. As expected, increased differences were observed for mixture spectra (Figure 2D–F, Figures S17–S20) due to increased sample complexity and a higher probability for competitive ionization. To better understand the global reproducibility of the mass spectra generated in the study, pairwise comparisons were conducted (comparing only spectra from the same solution) for all spectra collected by the same operator, the same lab but different operators (when available), and different laboratories across all three fragmentation levels (Figure 3, Table 3).

**Figure 3:**
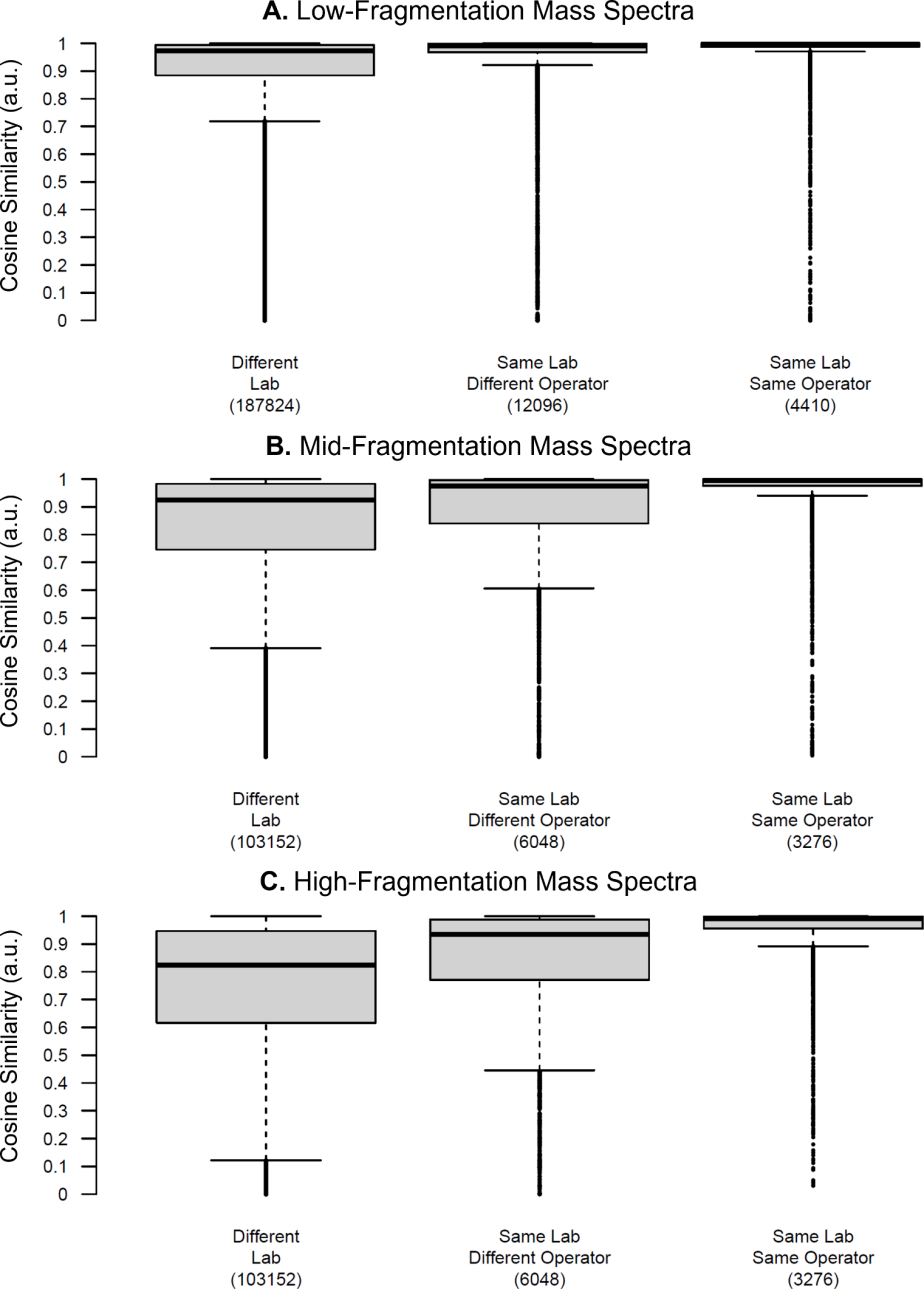
Distribution of all computed similarity scores of spectra collected of the same sample visualized as a box-and-whisker plot at (A) low-fragmentation energy, (B) mid-fragmentation energy, and (C) high-fragmentation energy, disaggregated by whether the spectra being compared were collected at (left) different laboratories, (center) the same lab but by different operators, or (right) the same operator. Values in parentheses below each box-and-whisker plot indicate the number of comparisons for that category. Similar plots for each of the 21 samples considered in this study are shown in Figures S21–S27. Note that several datafiles that were corrupt or empty and, therefore, aggregated mass spectra could not be obtained.

**Table 3:** Median and Lower Quartile Pairwise Cosine Similarity Values for the Box-and-Whisker Plots Shown in Figure 3

	**Different Lab**	Same Lab, Different Operator	Same Lab, Same Operator
**Median (a.u.)**			
**Low-Fragmentation**	0.973	0.992	0.998
**Mid-Fragmentation**	0.923	0.975	0.996
**High-Fragmentation**	0.824	0.935	0.992

**Lower Quartile (a.u.)**			
**Low-Fragmentation**	0.884	0.968	0.988
**Mid-Fragmentation**	0.746	0.841	0.976
**High-Fragmentation**	0.617	0.771	0.955

As is to be expected, the cosine similarity scores were the highest for pairwise comparisons of mass spectra collected from the same operator. The lower quartile of the computed similarity scores ranged from 0.955 a.u. to 0.988 a.u. (Table 3), depending on fragmentation level, indicating a high degree of spectral similarity for the majority of mass spectra. For laboratories with multiple operators, the low-fragmentation mass spectra, commonly dominated by the intact protonated molecule of the analyte(s), remained highly reproducible, with a median cosine similarity of 0.992 a.u. and a lower quartile of 0.968 a.u. Reproducibility at the mid- and high-fragmentation energies was slightly lower than that of the same operator comparisons, indicating that there may be operator-dependent factors driving reproducibility. These factors include how samples are introduced into the system, how long samples are kept in the gas stream (for manual DART-MS analysis), and perfumes or body lotions that may influence or contribute to the mass spectra. Pairwise comparisons of spectra generated across laboratories were the least reproducible, although the median and lower quartile cosine similarity scores were still impressive, given that these comparisons spanned different instrument types (high- and low-resolution), different AI sources, different analytical methods, and different environmental conditions. As was observed with the same lab but different operator comparisons, reproducibility was higher for the low-fragmentation mass spectra than the mid- and high-fragmentation mass spectra. This likely reflects a combination of the factors mentioned above along with variability in the *is*-CID energies used across laboratories. Since *is*-CID occurs in the inlet of the mass spectrometer, differences in ion optics between systems also influence the resulting spectra.

#### 3.2.2 Comparison of Lab and Operator Reproducibility Indices

While understanding reproducibility across laboratories is extremely important, especially as it relates to the use of centrally curated spectral libraries (the focus of future work), from a forensic science context it is important to demonstrate comparable measurements to ensure consistency, reliability, and reproducibility of results within a laboratory system. Lab and operator reproducibility indices were calculated for all 21 solutions analyzed. As stated previously, the operator reproducibility index (ORI) is the lowest cosine similarity score obtained from pairwise comparisons of mass spectra collected by a unique operator analyzing a particular solution. Similarly, the lab reproducibility index (LRI) is the lowest cosine similarity score obtained from pairwise comparisons of mass spectra collected by different operators in the same lab analyzing a particular solution.

ORIs varied significantly between operators, both globally and within a laboratory (Figure 4). The two issues identified in the visual examination of the heatmaps were in the ORI plots for Operators 1-1, 3-1, and 3-2 (Figure 4). For Operator 1-1, the low ORIs for the compounds analyzed earlier within a measurement session (compounds were largely analyzed in the order presented in the legend) highlighted the persistent carryover of PEG within the system (as discussed above) for at least the beginning of the measurement session, leading to lower ORIs. Similarly, ORIs for the later compounds and the mixtures showed high cosine similarity values, indicating that the PEG carryover had likely thermally desorbed by this time. For Operators 3-1 and 3-2, a wide range of ORIs were obtained, consistent with the sporadic sample introduction and incomplete thermal desorption of the analyte observed in Figure 2.

**Figure 4:**
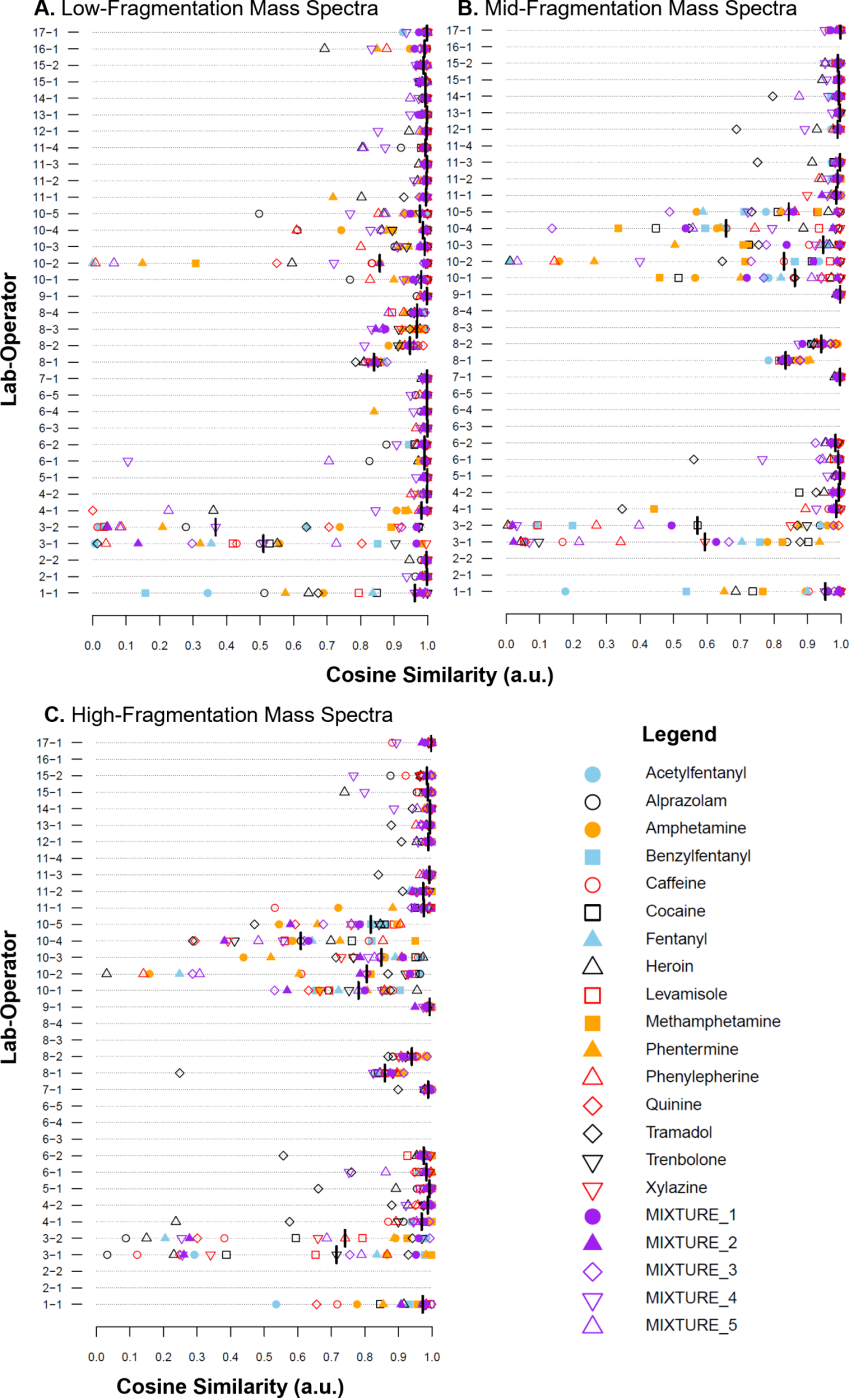
Operator reproducibility indices (ORIs) for each of the 21 solutions (color and type of point). Spectra were measured at (A) low-fragmentation energy, (B) mid-fragmentation energy, or (C) high-fragmentation energy. Note that not all operators took measurements at mid and high *is*-CID energies. The black bar is the median reproducibility index for the operator.

In addition to those observations, the variability of ORIs from operators within a laboratory was interesting. For example, all operators in laboratories 6 and 15 consistently obtained high ORIs across different samples. In contrast, lab 10 showed a wider spread in ORIs among operators despite using the same method. Investigation of the heat maps (Figures S2–S20) showed that there appeared to be a measurement session for Operator 10-2 that produced noisier mass spectra than any other session. Lab 8 was also interesting in that each operator appeared to have a narrow range of ORIs but at different median values. For Operator 8-1, all ORIs are grouped together at a value of ∼0.85 a.u., indicating that there may have been a single measurement session that was slightly different than the others. Visual inspection of the heat maps showed no obvious differences.

As observed with the overall comparisons in the previous section, ORIs were generally lower for mid- and high-fragmentation spectra relative to low-fragmentation spectra (Figure 4). For lab 10, a significant decrease in ORIs was observed at these higher energy levels, driven by a visually observable increase in noise in mass spectra when looking at the heat maps for these *is-*CID values. This could indicate a mass spectrometer inlet that needs cleaning, which produces artifact signals and inconsistent fragmentation. Possible inconsistencies with high-energy *is-*CID spectra can be observed with highly reproducible laboratories, such as laboratories 7, 9, 14, and 15 (Figure 4), all of which produced at least one ORI significantly lower than the median for the high-fragmentation mass spectra.

Another key takeaway from the ORI analysis is that ORIs values do not appear to be strongly dependent on compound identity, with one exception. The 16 single-compound solutions were chosen to represent a range of compound classes, polarities, and molecular masses to evaluate whether the compound type affects mass spectral reproducibility. However, since ORIs varied across operators and fragmentation levels, and some laboratories consistently produced highly reproducible spectra regardless of the compound type, no clear relationship was observed, at least within the scope of compounds tested. Similarly, ORIs for mixture spectra (Figure 4, purple points) were not consistently lower than ORIs for single-component spectra, indicating that sample complexity does not significantly impact reproducibility under the conditions evaluated here.

The one compound that did prove to be possibly problematic was tramadol (Figure 4, black hollow diamonds). Tramadol has been noted to be problematic in previous work^22^ due to the fact that, at high *is-*CID energies, it will fragment almost exclusively into an ion at nominal *m*/*z* 58. Many laboratories did not scan below *m*/*z* 80 or *m*/*z* 60 in order to avoid atmospheric background signals. In these instances, comparisons are being conducted on spectra largely made up of noise (since the *m*/*z* 58 ion would not be present), leading to low cosine similarity values.

A final observation from the ORI plot is that, although there are limited data, the use of different ionization sources or mass spectrometers did not drive reproducibility trends. Lab 15 used the ASAP source (while all others used DART) and produced data as reproducible as those from many of the laboratories using DART. Similarly, the mass spectrometer type did not affect reproducibility. Laboratories 1–10, 12, and 13 (and Operators 11-1 to 11-3) used JEOL AccuTOF mass spectrometers, whereas laboratories 14–17 (and Operator 11-4) used other mass spectrometer brands.

The LRIs (Figure 5) showed largely the same trends as the ORI plot (Figure 4). For laboratories where ORIs were consistent across operators (laboratories 2, 4, 6, 8, and 15), LRIs reflected similar trends. Interestingly, for lab 3, LRIs were generally lower for low-fragmentation mass spectra compared to mid- and high-fragmentation, which was opposite of what has been observed previously. This could be due to higher noise in the low-fragmentation spectra, which is collapsed into fewer ions at higher *is-*CID energies. It should be noted that the drop in LRIs for lab 11 is likely driven by the fact that three different mass spectrometers were used across the four operators, unlike other laboratories where the same instrument was used regardless of operator.

**Figure 5:**
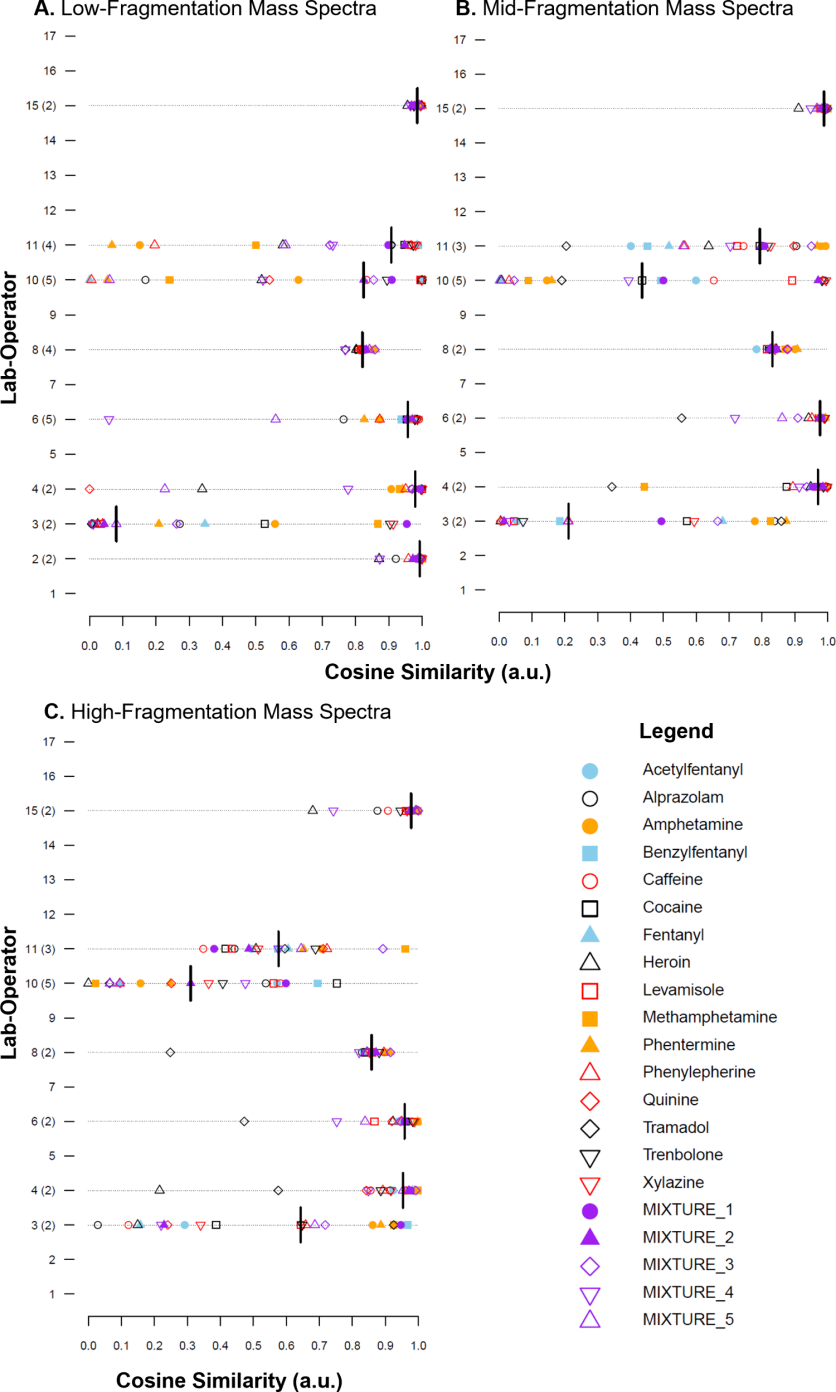
Laboratory reproducibility indices (LRIs) for each of the 21 solutions (color and type of point). Spectra were measured at (A) low-fragmentation energy, (B) mid-fragmentation energy, or (C) high-fragmentation energy. Note that not all laboratories have multiple operators. The black bars are the median reproducibility index for the laboratory.

A final comparison of ORIs was made to determine whether other non-method-specific metrics, such as operator experience, instrument age, and building age, affected mass spectral reproducibility. Operator experience was investigated to see whether those new to using AI-MS produced results that were less reproducible than those with significant experience, which could be critical for manual sample introduction. Instrument age was investigated to see if reproducibility could be affected as the instruments aged. Since AI-MS systems are sensitive to environmental surroundings, building age was also investigated since new buildings could be off-gassing chemicals that interfere with AI-MS data collection or old buildings may not have the ventilation needed to remove vapors (e.g., solvent vapors) that could affect the signal. Comparison of ORIs for the operators across these three metrics, using the provided survey data (Figure 6), showed no obvious trends for any of these potential contributors. The few operators with lower ORIs all have less than three years of experience using AI-MS, indicating a possible correlation between experience and reproducibility. This is further supported by the observation that all operators with more than three years of experience had ORIs above 0.9 a.u. However, there were many operators with less than three years of experience that still had high ORIs. Instrument age showed no correlation with ORI, indicating that instrument-related variability is likely linked to instrument cleanliness and method-specific parameters. Building age also showed no obvious correlation. Although the lower ORIs were observed exclusively in newer buildings (<5 years), both cases also involved operators with less than three years of experience, suggesting that operator experience was the primary contributing factor.

**Figure 6:**
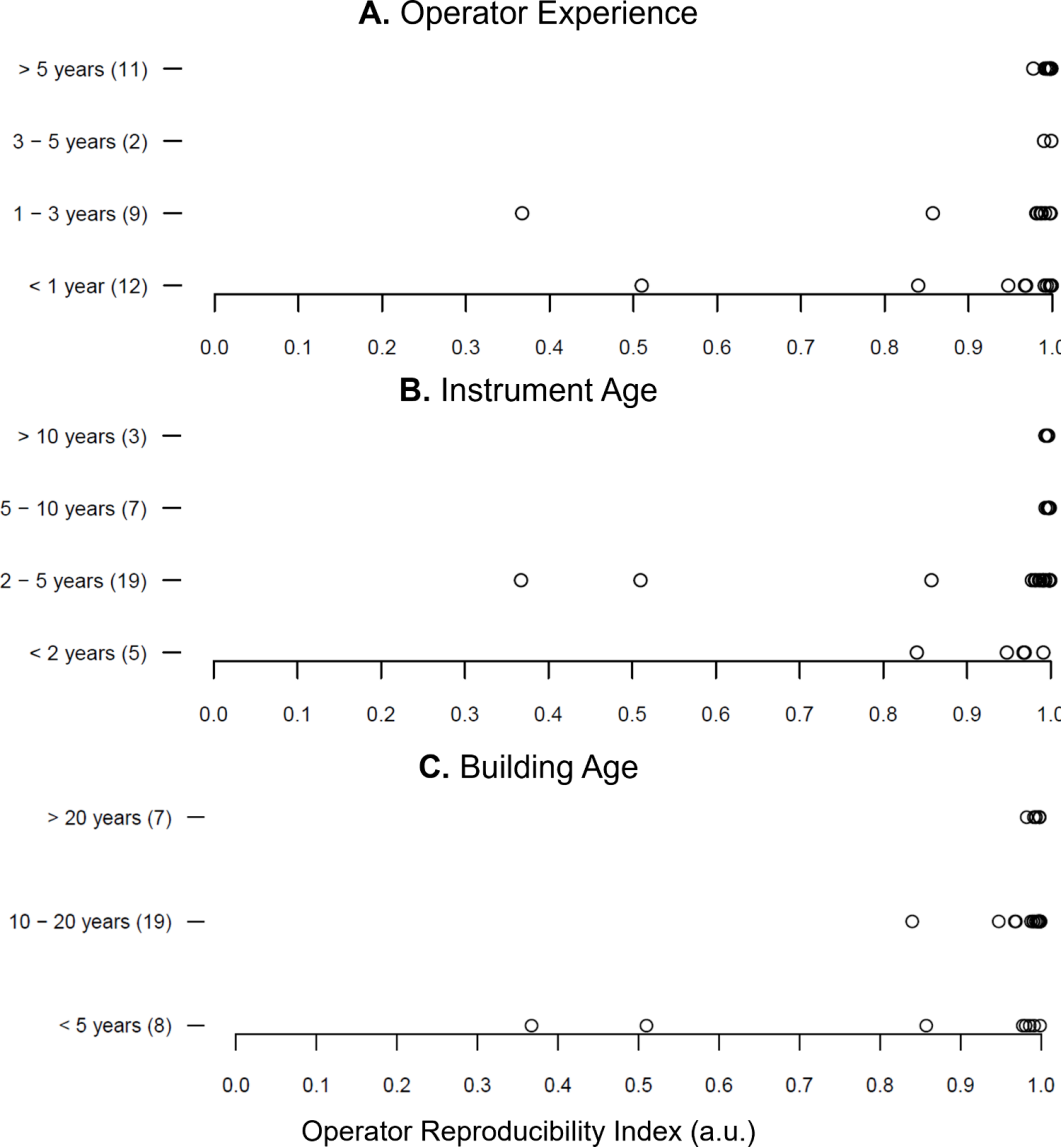
Operator reproducibility index (ORI) as a function of (A) operator experience, (B) instrument age, and (C) building age. The number in parentheses is the number of responses for that category.

#### 3.2.3 Potential Benefits of Using a Uniform Analytical Method

The final component of the study sought to address whether using uniform analytical methods improved the reproducibility of mass spectra across laboratories. Since this study component was optional and required the operator to use a JEOL AccuTOF mass spectrometer, only five operators participated (7-1, 11-1, 11-2, 12-1, and 13-1). Operators were requested to complete two measurement sessions using the prescribed methods (Table S2) in addition to the four measurement sessions they had already completed using their own methods. Pairwise comparisons were then performed between spectra generated by the same operator and those generated by different operators.

As expected, whether an operator used their own method or the uniform method (Figure 7, right) did not dramatically affect the pairwise cosine similarity scores for the same operator comparisons. For the same operator data, the uniform method produced slightly higher median and lower quartile similarity scores compared to their own method (Table 4). However, these minor differences are unlikely to impact downstream data interpretation.

**Figure 7:**
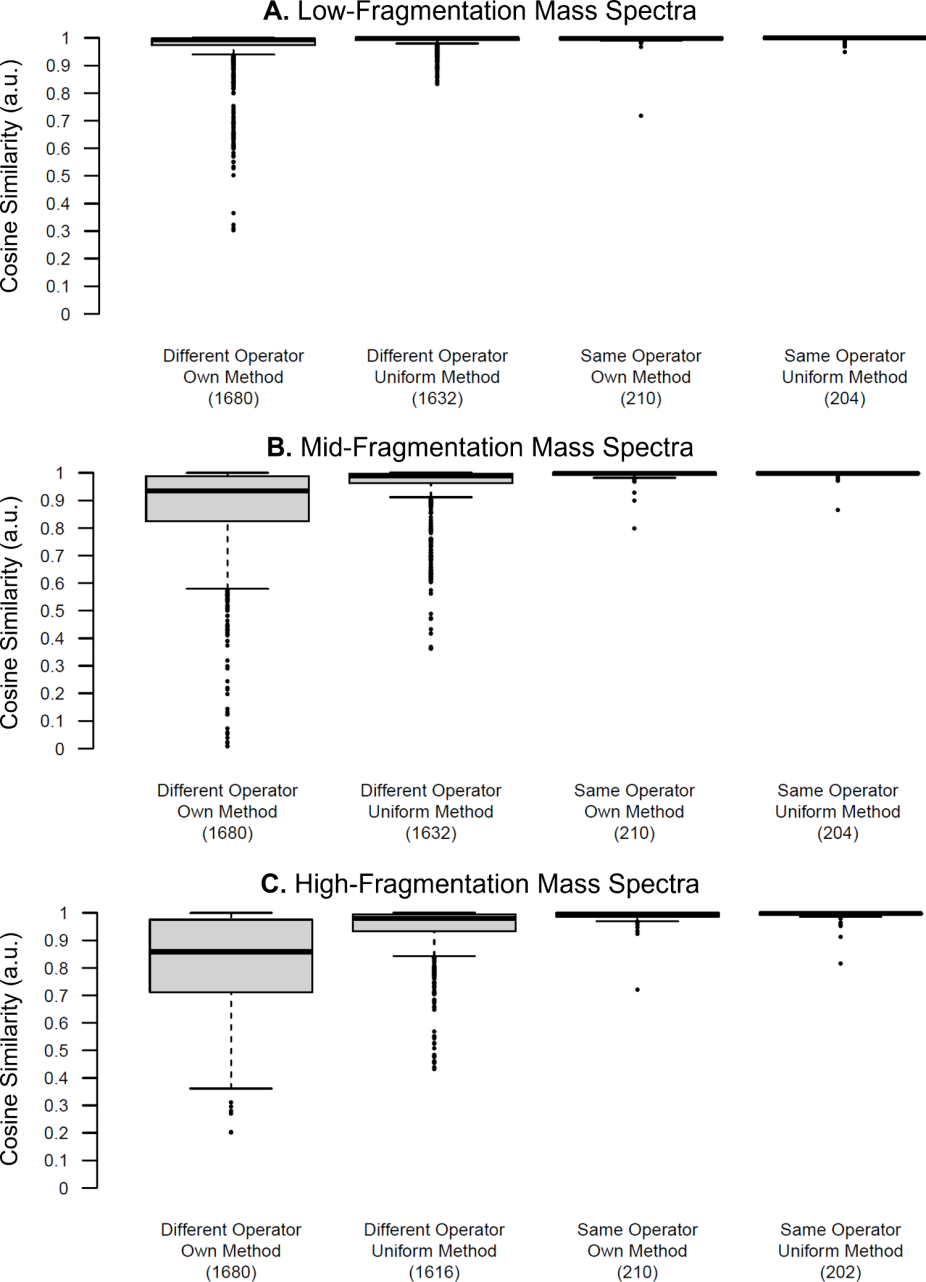
All computed similarity scores of spectra collected of the same sample, visualized as a box-and-whisker plot, disaggregated by whether the spectra being compared were collected by different operators (left) or the same operator (right) using either their own method or the uniform method at (A) low-fragmentation, (B) mid-fragmentation, and (C) high-fragmentation energies. Values in parentheses below each box-and-whisker plot indicate the number of comparisons for that category. Note that several datafiles were corrupt or empty and, therefore, aggregated mass spectra could not be obtained.

**Table 4:** Median and Lower Quartile Pairwise Cosine Similarity Values for the Box-and-Whisker Plots Shown in Figure 7

	Different Operator, Own Method	Different Operator, Uniform Method	Same Operator, Own Method	Same Operator, Uniform Method
**Median (a.u.)**				
**Low-Fragmentation**	0.992	0.998	0.999	0.999
**Mid-Fragmentation**	0.934	0.990	0.998	0.999
**High-Fragmentation**	0.859	0.979	0.995	0.998

**Lower Quartile (a.u.)**				
**Low-Fragmentation**	0.974	0.991	0.996	0.998
**Mid-Fragmentation**	0.824	0.923	0.992	0.996
**High-Fragmentation**	0.712	0.933	0.987	0.994

More substantial differences were obtained when comparing data collected by different operators. Similar to the data discussed previously, minor differences were seen in low-fragmentation data (Figure 7, top left, and Table 4) since these spectra are dominated by intact protonated molecules. At mid-fragmentation and high-fragmentation levels, a significant improvement in mass spectral reproducibility was observed when different operators used the uniform method. The lower quartile similarity scores improved from 0.82 to 0.92 a.u. for mid-fragmentation spectra and from 0.71 to 0.93 a.u. for high-fragmentation spectra. These improvements indicate that the majority of spectra collected using the uniform method had pairwise similarity scores above 0.9 a.u., indicating a high degree of similarity. The gains in mass spectral reproducibility when using uniform method parameters could have significant impacts when using centrally curated mass spectral libraries or when data from multiple laboratories are used for data mining, sample linking, or other forensic intelligence applications. Heat maps for all spectra collected using the uniform method can be found in Figures S28–S38.

## 4 Conclusion

The goal of this paper was to address three target questions through an interlaboratory study focused on ambient ionization mass spectrometry for seized drug analysis:

(1) What methods and instrumentation are used in seized drug laboratories in the United States?
(2) What is the intraperson, intralaboratory, and interlaboratory mass spectra variability for compounds and mixtures of relevance to seized drug analysis, and what factors drive variability differences?
(3) Does the use of predefined method parameters drive down interlaboratory mass spectral variability?

Considering the number of participants in this study, it is clear that AI-MS has established a significant presence in forensic laboratories for seized drug screening. Most laboratories in the study utilized a DART ionization source coupled with a time-of-flight mass spectrometer, although a variety of mass spectrometry systems are deployed. Many laboratories are using *is*-CID to obtain mass spectra at multiple fragmentation levels to enhance compound identification efforts. Manual sample introduction with use of helium as the ionization gas remains commonplace for DART-based analyses; however, with advancements in autosampler technology and the challenges of purchasing helium gas, it will be interesting to see how these metrics change over time. In terms of non-instrument metrics, operator experience was nearly equally distributed among less than one year experience, one to five years of experience, and greater than five years of experience, indicating that the adoption of AI-MS into laboratories is still ongoing. Of the 35 operators, roughly one-third reported not using a fume extractor or other ventilation system to remove vapors generated by AI-based sources, which is potentially concerning, given the risk of exposure to toxic substances when desorbing samples in ambient conditions.

Mass spectral variability was observed within and across laboratories; however, these values were better than expected, given the wide range of instruments, methods, and operators. Select instances of poorer than average reproducibility were identified and appeared to be driven by (1) carryover of calibration compounds, (2) poor sample introduction, or (3) instruments needing inlet cleaning. Mass spectral variability was not driven by compound type, MS type, MS age, operator experience, or building age. Variability was higher when comparing data collected from different operators; however, multiple laboratories demonstrated that obtaining highly reproducible data from a laboratory, even when using manual sample introduction, was possible. As expected, variability increased at mid- and high-fragmentation energies, given that *is-*CID relies on indiscriminate collisions of ions in the inlet of the mass spectrometer and therefore will be inherently more variable than tandem mass spectrometers.

Finally, it was demonstrated that mass spectral reproducibility increased when uniform analytical methods were used, especially at mid-fragmentation and high-fragmentation levels. Driving toward higher interlaboratory reproducibility is critical for multilaboratory systems, laboratories using centrally curated databases, and laboratories wanting to aggregate data to address challenges like data mining, sample linkage, source attribution, or other forensic intelligence questions.

It is important that we note several of the limitations present within the study. First, due to the high number of datafiles, all mass spectra were autoextracted based on operator-reported run times. Spot checks were completed, but it is possible that some of the carryover observed was due to incorrect run times noted. The order in which samples were run was neither prescribed nor randomized. By design, operators ran prepared solutions with known identities, and in doing so we are unable to measure any effect on variability from sample preparation. This will be the focus of a future study. We also did not ask operators to interpret the data, as the goal was to understand instrument performance differences before introducing the human interpretation factor. This may indicate that some operators did not review the data prior to submission and that issues such as carryover or poor peak heights might have been identified during normal operation.

While this study provides the foundation for a comparison of AI-MS data across seized drug laboratories, there are additional questions that remain to be answered. Future work will be focused on building upon this work to better understand the benefits and drawbacks of centrally curated spectral databases versus in-house databases for compound identification. In addition, this study purposefully did not consider the human interpretation component of data analysis, which warrants future study. Interpretation of the data likely also relies on a better understanding of sensitivity differences across laboratories and whether competitive ionization effects are universally observed. Through continuation of interlaboratory studies such as these, we can better understand the strengths and limitations of AI-MS approaches for seized drug screening and enable the development of evidence-based standards and error rates, key needs for the community as we seek to increase objectivity in forensic science.

## Supplementary Material



## Data Availability

All extracted mass spectra and example interlaboratory instructions and documentation are available upon request from the authors.

## Notes

Prestudy survey responses and method information for all participants; uniform method parameters; examples of chronograms that led to more variable mass spectral data; mass spectral heat maps for all compounds and mixtures not shown in the manuscript (including spectra collected using the uniform method); box-and-whisker plots comparing same operator, same lab, and different lab spectral similarities for all solutions (PDF)
