# Supplementary material for: Capturing Current Practices and Characterizing Measurement Reproducibility for Seized Drug Analysis Using Ambient Ionization Mass Spectrometry: An Interlaboratory Study
Source: J Am Soc Mass Spectrom. 2025 Aug 27;36(10):2239–52. doi: 10.1021/jasms.5c00213 (PMC12492400; doi:10.1021/jasms.5c00213)
Supplement: Supplementary file 1 [file js5c00213_si_001.pdf]

Supporting Information for:

Capturing Current Practices and Characterizing Measurement Reproducibility for Seized Drug Analysis using Ambient Ionization Mass Spectrometry – an Interlaboratory Study

Edward Sisco[a*], Dennis Leber[a], Arun S. Moorthy[b]

[a]National Institute of Standards and Technology, Gaithersburg, MD 20899, USA
[b]Department of Forensic Science, Trent University, Peterborough, ON K9G 0L2, Canada

[*]Corresponding Author Email: edward.sisco@nist.gov

Table S1. Pre-study survey responses and method information reported by study participants.

Operator	Uniform Method Operator	AI Source	MS Type	Experience (yr.)	Instrument Age (yr.)	Lab Age (yr.)	Fume Extractor?	Solvent Nearby?
1-1	--	DART	JEOL AccuTOF	N/A	N/A	N/A	N/A	N/A
2-1	--	DART	JEOL AccuTOF	5+	5-10	10-20	No	No
2-2	--	DART	JEOL AccuTOF	<1	5-10	10-20	No	No
3-1	--	DART	JEOL AccuTOF	<1	2-5	5-10	Yes	No
3-2	--	DART	JEOL AccuTOF	1-3	2-5	5-10	Yes	No
4-1	--	DART	JEOL AccuTOF	1-3	2-5	20+	No	No
4-2	--	DART	JEOL AccuTOF	1-3	2-5	20+	No	No
5-1	--	DART	JEOL AccuTOF	5+	2-5	10-20	No	No
6-1	--	DART	JEOL AccuTOF	3-5	2-5	10-20	Yes	No
6-2	--	DART	JEOL AccuTOF	<1	2-5	10-20	Yes	No
6-3	--	DART	JEOL AccuTOF	<1	2-5	10-20	Yes	No
6-4	--	DART	JEOL AccuTOF	<1	2-5	10-20	Yes	No
6-5	--	DART	JEOL AccuTOF	<1	2-5	10-20	Yes	No
7-1	1	DART	JEOL AccuTOF	3-5	5-10	5-10	No	No
8-1	--	DART	JEOL AccuTOF	<1	0-2	10-20	Yes	No
8-2	--	DART	JEOL AccuTOF	<1	0-2	10-20	Yes	No
8-3	--	DART	JEOL AccuTOF	<1	0-2	10-20	Yes	No
8-4	--	DART	JEOL AccuTOF	<1	0-2	10-20	Yes	No
9-1	--	DART	JEOL AccuTOF	<1	2-5	10-20	Yes	No
10-1	--	DART	JEOL AccuTOF	1-3	2-5	5-10	Yes	No
10-2	--	DART	JEOL AccuTOF	1-3	2-5	5-10	Yes	No
10-3	--	DART	JEOL AccuTOF	1-3	2-5	5-10	Yes	No
10-4	--	DART	JEOL AccuTOF	1-3	2-5	5-10	Yes	No

10-5	--	DART	JEOL AccuTOF	5+	2-5	5-10	Yes	No
11-1	2	DART	JEOL AccuTOF	5+	10+	10-20	Yes	No
11-2	3	DART	JEOL AccuTOF	1-3	10+	10-20	Yes	No
11-3	--	DART	JEOL AccuTOF	5+	5-10	10-20	Yes	No
11-4	--	DART	Thermo Q-Exactive	5+	10+	20+	No	Yes
12-1	4	DART	JEOL AccuTOF	5+	5-10	10-20	Yes	No
13-1	5	DART	JEOL AccuTOF	5+	5-10	20+	No	No
14-1	--	DART	Thermo Fortis	5+	5-10	20+	N o	Y es
15-1	--	ASAP	Waters RADIAN	<1	2-5	10-20	No	N/A
15-2	--	ASAP	Waters RADIAN	1-3	2-5	10-20	No	N/A
16-1	--	DART	Thermo Fortis	5+	0-2	20+	No	Yes
17-1	--	DART	Waters QDa	5+	2-5	20+	Yes	Yes

Table S1, continued. Pre-survey responses and method information reported by study participants.

Operator	Mass Calibrant	Mass Calibration Frequency	Positive Control Compound	Positive Control Frequency	Negative Control	Negative Control Frequency
1-1	PEG	With Sample	PEG	With Sample	N/A	N/A
2-1	PEG	With Sample	Linoleic Acid	With Sample	N/A	N/A
2-2	PEG	With Sample	Linoleic Acid	With Sample	N/A	N/A
3-1	PEG	Weekly	Caffeine	Daily	N/A	N/A
3-2	PEG	Weekly	Caffeine	Daily	N/A	N/A
4-1	PEG	Daily	Caffeine	With Sample	N/A	N/A
4-2	PEG	Daily	Caffeine	With Sample	N/A	N/A
5-1	PEG	With Sample	Custom Mix	With Sample	Solvent Blank	Only w/ Pharm ID
6-1	PEG	Weekly	Cocaine	With Sample	Tetracaine	With Sample
6-2	PEG	Weekly	Cocaine	With Sample	Tetracaine	With Sample
6-3	PEG	Weekly	Cocaine	With Sample	Tetracaine	With Sample
6-4	PEG	Weekly	Cocaine	With Sample	Tetracaine	With Sample
6-5	PEG	Weekly	Cocaine	With Sample	Tetracaine	With Sample
7-1	PEG	With Sample	Custom Mix	With Sample	Solvent Blank	With Sample
8-1	PEG	Weekly	Cocaine	Daily	Tetracaine	With Sample
8-2	PEG	Weekly	Cocaine	Daily	Tetracaine	With Sample
8-3	PEG	Weekly	Cocaine	Daily	Tetracaine	With Sample
8-4	PEG	Weekly	Cocaine	Daily	Tetracaine	With Sample
9-1	PEG	With Sample	Mannitol	With Sample	Solvent Blank	With Sample
10-1	PEG	Weekly	Caffeine	With Sample	N/A	N/A
10-2	PEG	Weekly	Caffeine	With Sample	N/A	N/A
10-3	PEG	Weekly	Caffeine	With Sample	N/A	N/A
10-4	PEG	Weekly	Caffeine	With Sample	N/A	N/A
10-5	PEG	Weekly	Caffeine	With Sample	N/A	N/A
11-1	PEG	Daily	AB-FUBINACA	With Sample	Glass Rod	With Sample
11-2	PEG	Daily	AB-FUBINACA	With Sample	Glass Rod	With Sample
11-3	PEG	Daily	AB-FUBINACA	With Sample	Glass Rod	With Sample
11-4	Custom Mix	Weekly	N/A	With Sample	N/A	N/A
12-1	PEG	With Sample	Custom Mix	With Sample	Solvent Blank	Only w/ Pharm ID
13-1	PEG	With Sample	Custom Mix	With Sample	Solvent Blank	With Sample
14-1	Pierce EMRS	Weekly	N/A	N/A	Glass Rod	With Sample
15-1	PEG	Daily	Quinine	Daily	Solvent Blank	With Sample
15-2	PEG	Daily	Quinine	Daily	Solvent Blank	With Sample
16-1	Pierce EMRS	Biannually	Custom Mix (2)	Once per Instrument	Solvent Blank	With Sample
17-1	PEG	N/A	Custom Mix (3)	Daily	PTFE Swab	With Sample

Table S1, continued. Pre-survey responses and method information reported by study participants.

Operator	DART Source Temp (°C)	DART Gas	Grid Voltage (V)	Low *m/z*	High *m/z*	# *is*-CID Voltages Scanned	*Is*-CID Levels (V)
1-1	400	He	350	80	800	4	10, 30, 60, 90
2-1	400	He	150	60	700	1	20
2-2	400	He	150	60	700	1	20
3-1	400	He	350	80	800	4	10, 30, 60, 90
3-2	400	He	350	80	800	4	10, 30, 60, 90
4-1	400	He	350	80	800	4	10, 30, 60, 90
4-2	400	He	350	80	800	4	10, 30, 60, 90
5-1	350	He	150	60	600	4	20, 30, 60, 90
6-1	400	He	150	50	800	3	30, 60, 90
6-2	400	He	150	50	800	3	30, 60, 90
6-3	400	He	150	80	800	1	30
6-4	400	He	150	80	800	1	30
6-5	400	He	150	80	800	1	30
7-1	350	He	50	66	600	4	20, 30, 60, 90
8-1	400	He	150	80	800	3	30, 60, 90
8-2	400	He	150	80	800	3	30, 60, 90
8-3	400	He	150	50	800	1	30
8-4	400	He	150	50	800	1	30
9-1	350	He	150	10	1000	4	20, 30, 60, 90
10-1	400	He	150	80	800	3	30, 60, 90
10-2	400	He	150	80	800	3	30, 60, 90
10-3	400	He	150	80	800	3	30, 60, 90
10-4	400	He	150	80	800	3	30, 60, 90
10-5	400	He	150	80	800	3	30, 60, 90
11-1	400	He	150	80	800	3	30, 60, 90
11-2	400	He	150	80	800	3	30, 60, 90
11-3	400	He	50	80	800	3	30, 60, 90
11-4	350	He	350	75	1125	1	N/A
12-1	350	He	150	66	600	4	20, 30, 60, 90
13-1	350	He	250	66	600	4	20, 30, 60, 90
14-1	300	N2	250	50	750	1	30
15-1	N/A	N/A	N/A	50	600	4	15, 25, 35, 50
15-2	N/A	N/A	N/A	50	600	4	15, 25, 35, 50
16-1	250	He	350	50	500	1	5
17-1	300	N2	300	50	1250	4	15, 30, 50, 70

Table S2. Method parameters for the uniform method component of the study.

DART	
Gas Type	Helium
Gas Temperature	400 °C
Ionization Mode	Positive
Exit Grid Voltage	150 V
**Mass Spectrometer**	
Orifice Temperature	120 °C
Orifice 1 Voltage	Switching
Ring Lens Voltage	+5 V
Orifice 2 Voltage	+5 V
Ion Guide Voltage	+800 V
Mass Scan Range	*m/z* 80 – *m/z* 800
Parameter Switching Enabled?	Yes
Orifice 1 Voltage Cycle	+30 V, +60 V, +90 V
Orifice 1 Cycle Scan Time	0.4 s/voltage

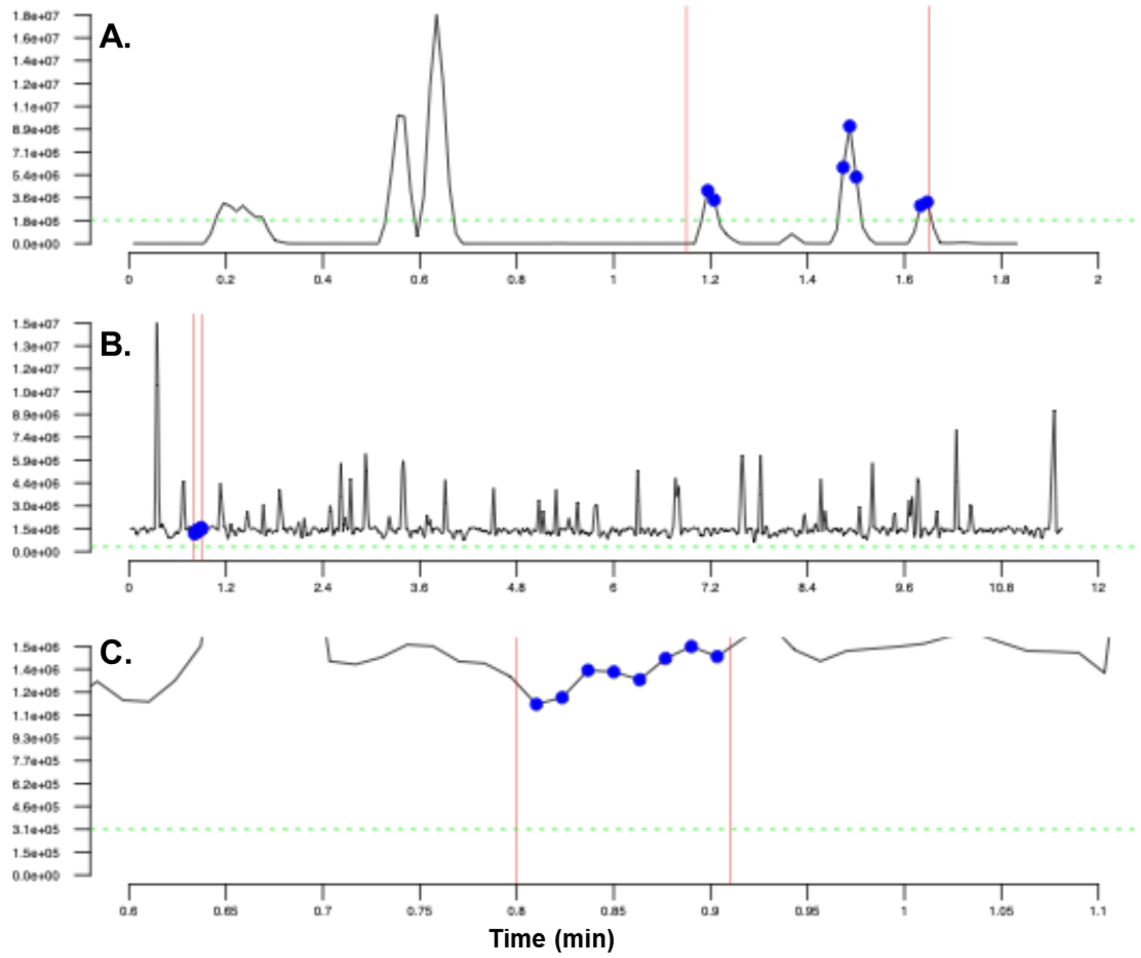

Figure S1. Examples of chronograms collected in the study. (A.) A chronogram collected following study protocols. (B.) Chronogram where all samples were measured sequentially in a single run (against protocol). (C.) Zoom in of chronogram from panel B., demonstrating no separation between sampling peak and background.

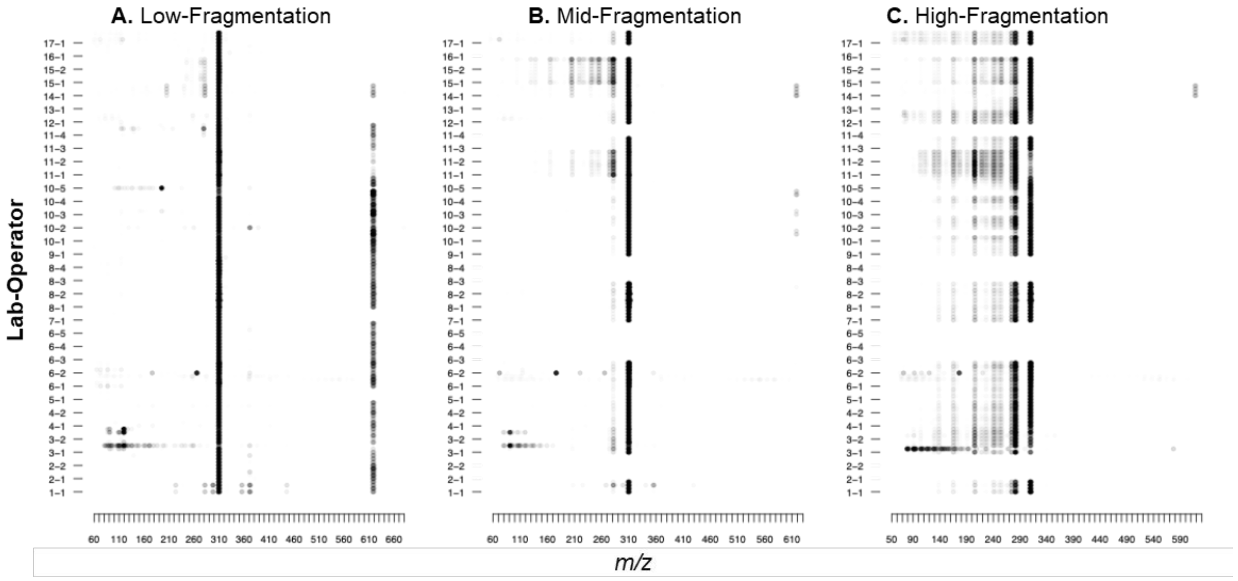

Figure S2. Example heat maps using aggregated is-CID mass spectral measurements of alprazolam at (A.) low-fragmentation energy, (B.) mid-fragmentation energy, and (C.) high-fragmentation energy. Each operator completed four measurement sessions, resulting in four aggregated spectra per operator. Note that not all operators provided measurements at the mid- and high-fragmentation levels.

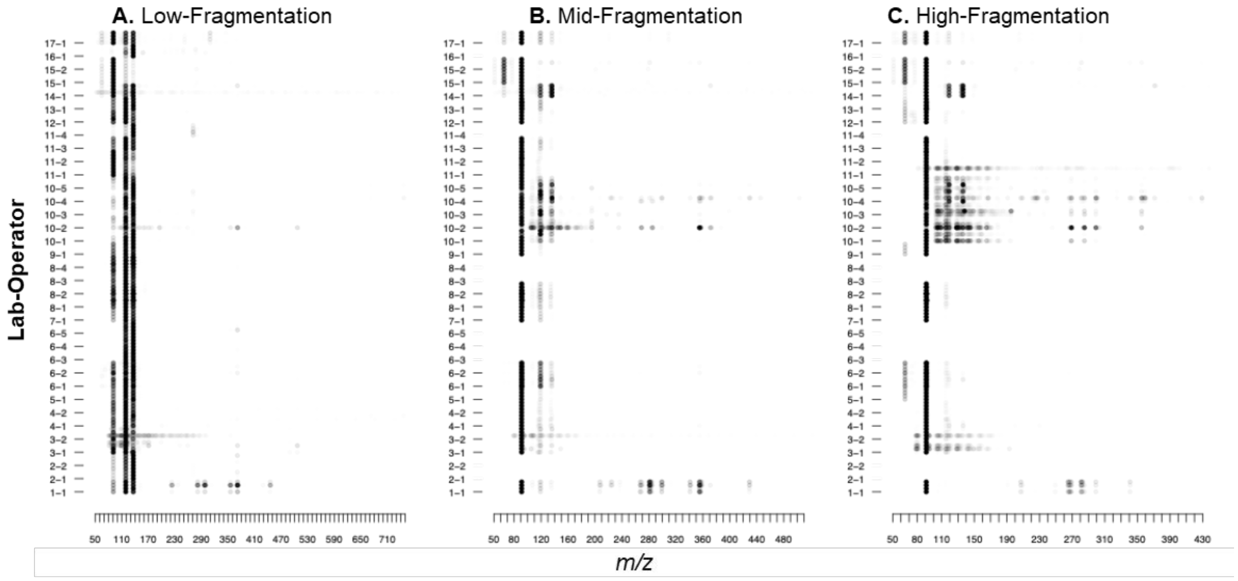

Figure S3. Example heat maps using aggregated is-CID mass spectral measurements of amphetamine at (A.) low-fragmentation energy, (B.) mid-fragmentation energy, and (C.) high-fragmentation energy. Each operator completed four measurement sessions, resulting in four aggregated spectra per operator. Note that not all operators provided measurements at the mid- and high-fragmentation levels.

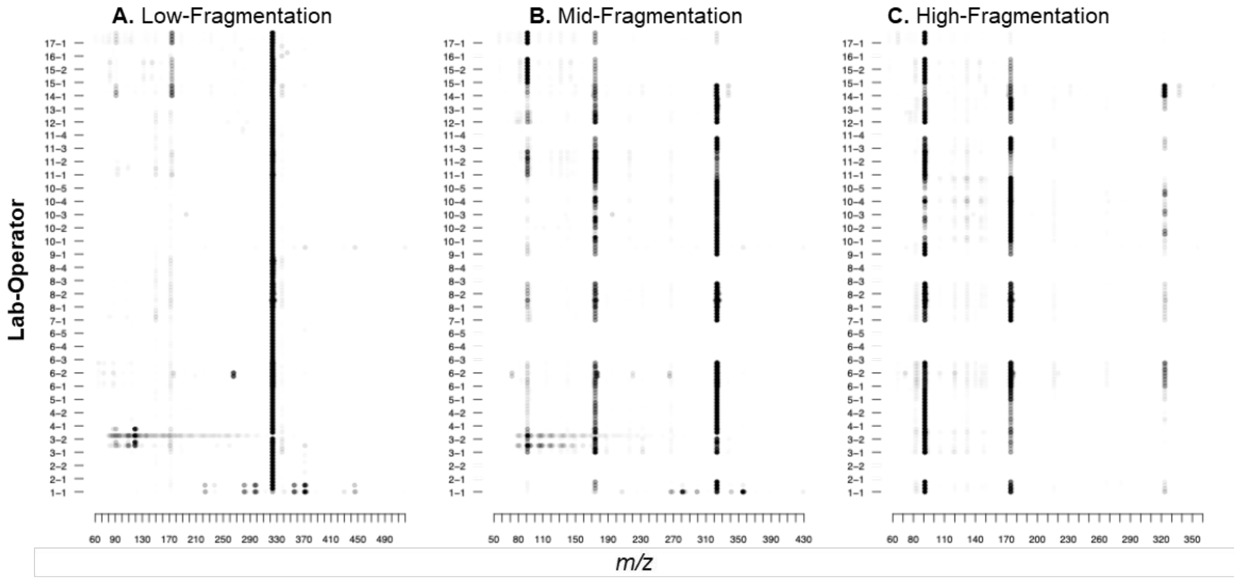

Figure S4. Example heat maps using aggregated is-CID mass spectral measurements of benzyl fentanyl at (A.) low-fragmentation energy, (B.) mid-fragmentation energy, and (C.) high-fragmentation energy. Each operator completed four measurement sessions, resulting in four aggregated spectra per operator. Note that not all operators provided measurements at the mid- and high-fragmentation levels.

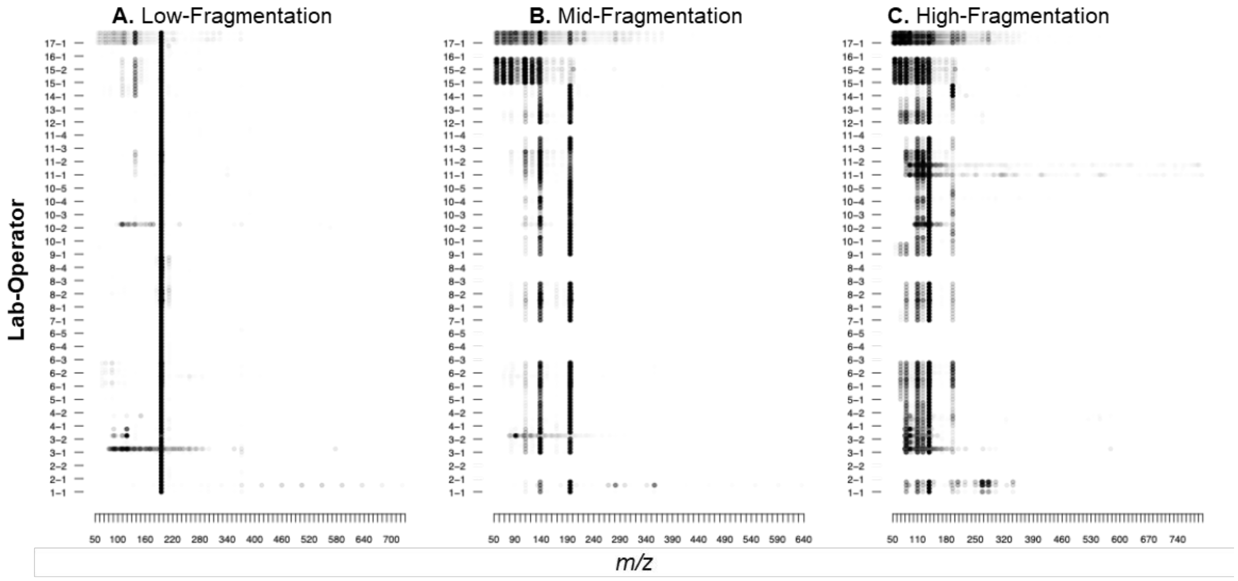

Figure S5. Example heat maps using aggregated is-CID mass spectral measurements of caffeine at (A.) low-fragmentation energy, (B.) mid-fragmentation energy, and (C.) high-fragmentation energy. Each operator completed four measurement sessions, resulting in four aggregated spectra per operator. Note that not all operators provided measurements at the mid- and high-fragmentation levels.

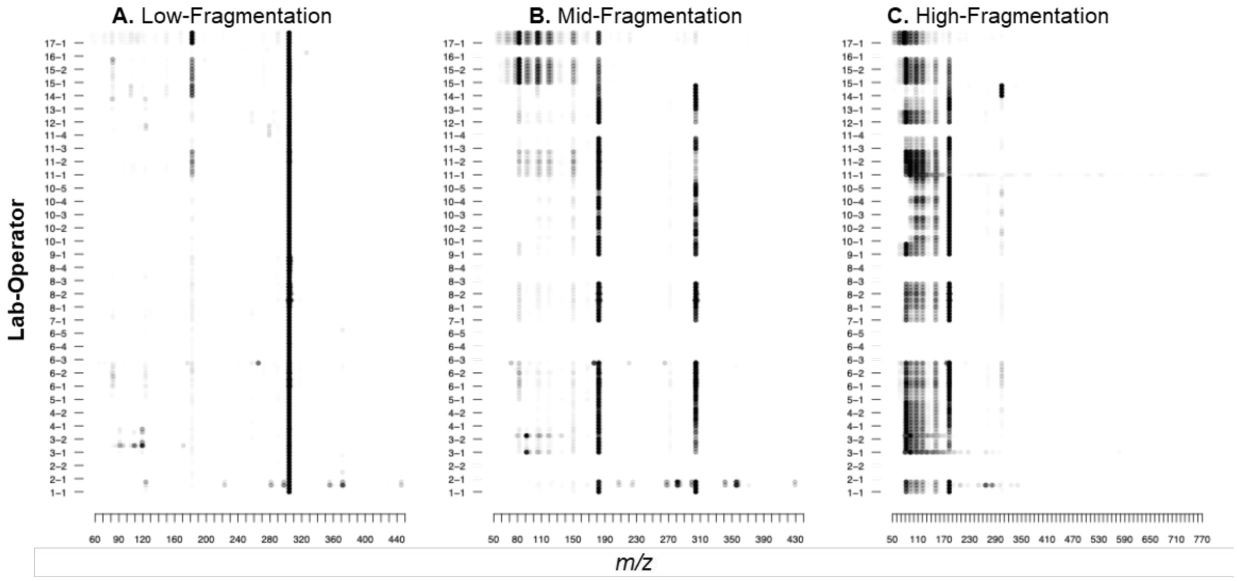

Figure S6. Example heat maps using aggregated is-CID mass spectral measurements of cocaine at (A.) low-fragmentation energy, (B.) mid-fragmentation energy, and (C.) high-fragmentation energy. Each operator completed four measurement sessions, resulting in four aggregated spectra per operator. Note that not all operators provided measurements at the mid- and high-fragmentation levels.

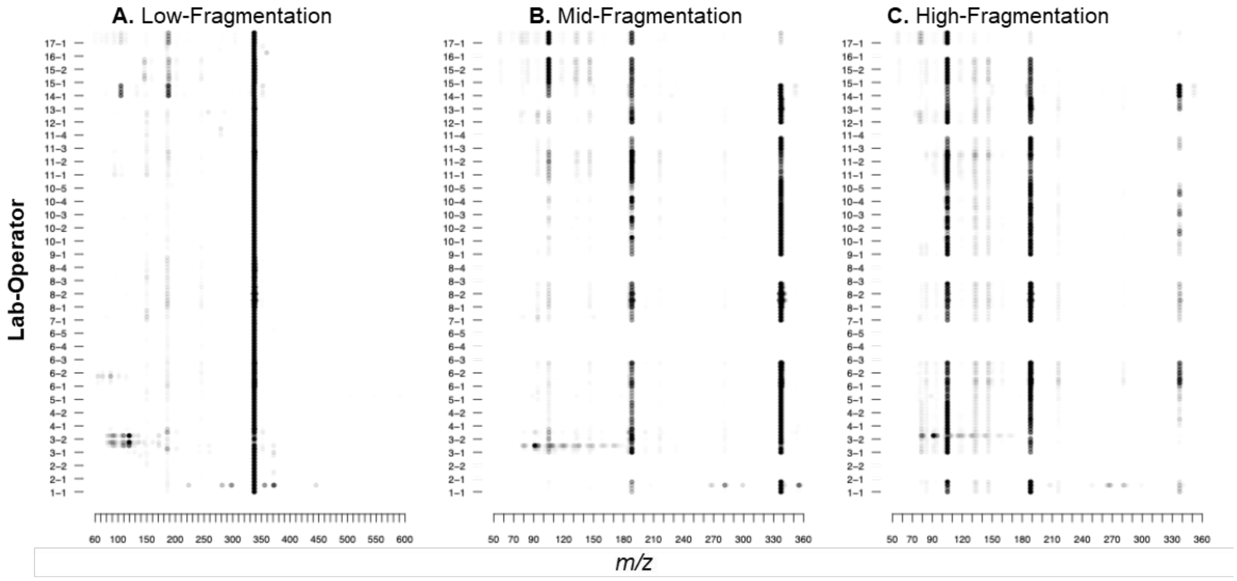

Figure S7. Example heat maps using aggregated is-CID mass spectral measurements of fentanyl at (A.) low-fragmentation energy, (B.) mid-fragmentation energy, and (C.) high-fragmentation energy. Each operator completed four measurement sessions, resulting in four aggregated spectra per operator. Note that not all operators provided measurements at the mid- and high-fragmentation levels.

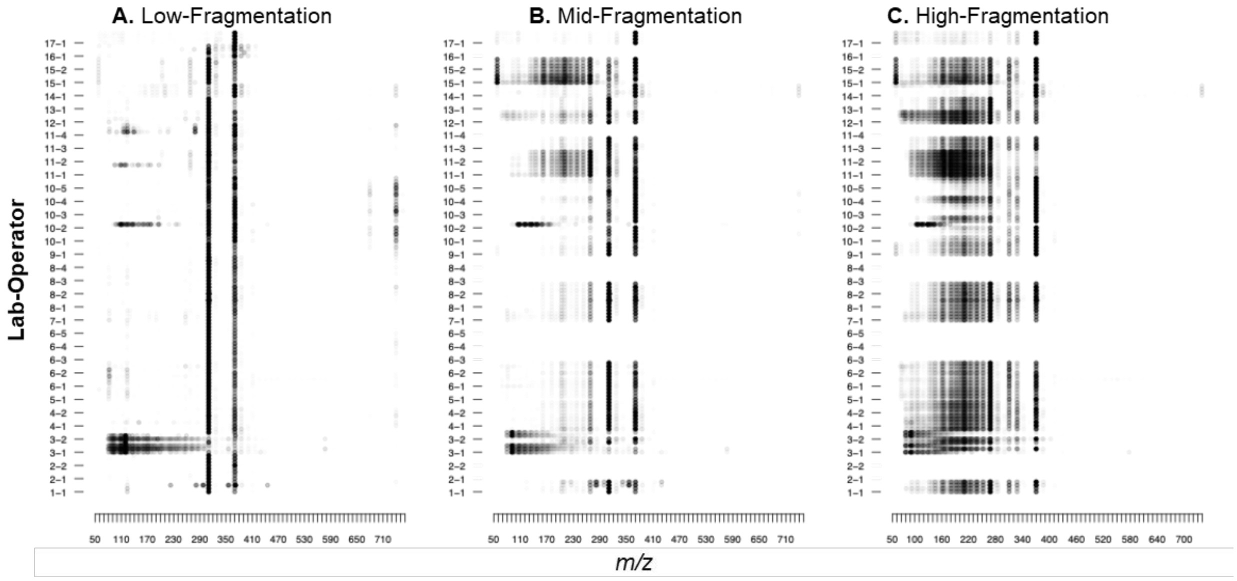

Figure S8. Example heat maps using aggregated is-CID mass spectral measurements of heroin at (A.) low-fragmentation energy, (B.) mid-fragmentation energy, and (C.) high-fragmentation energy. Each operator completed four measurement sessions, resulting in four aggregated spectra per operator. Note that not all operators provided measurements at the mid- and high-fragmentation levels.

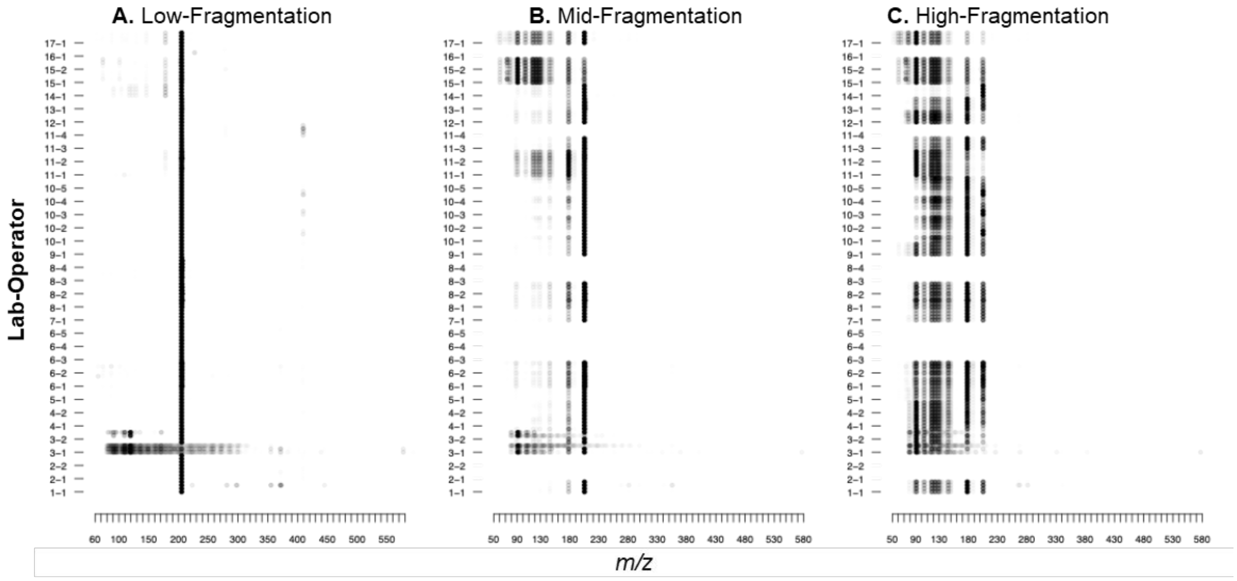

Figure S9. Example heat maps using aggregated is-CID mass spectral measurements of levamisole at (A.) low-fragmentation energy, (B.) mid-fragmentation energy, and (C.) high-fragmentation energy. Each operator completed four measurement sessions, resulting in four aggregated spectra per operator. Note that not all operators provided measurements at the mid- and high-fragmentation levels.

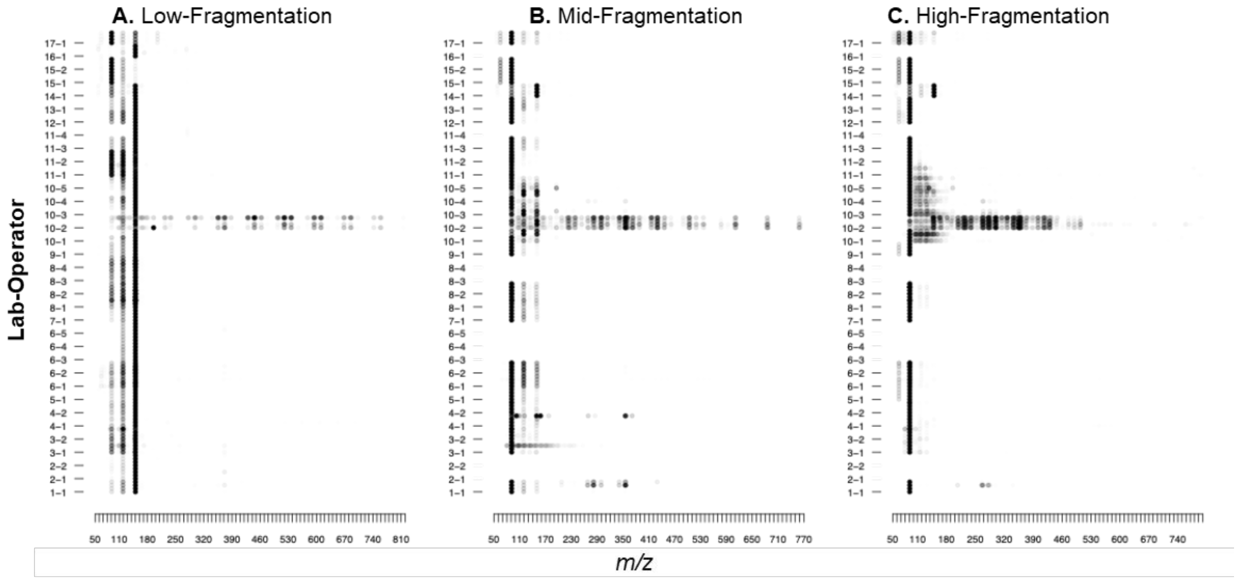

Figure S10. Example heat maps using aggregated is-CID mass spectral measurements of methamphetamine at (A.) low-fragmentation energy, (B.) mid-fragmentation energy, and (C.) high-fragmentation energy. Each operator completed four measurement sessions, resulting in four aggregated spectra per operator. Note that not all operators provided measurements at the mid- and high-fragmentation levels.

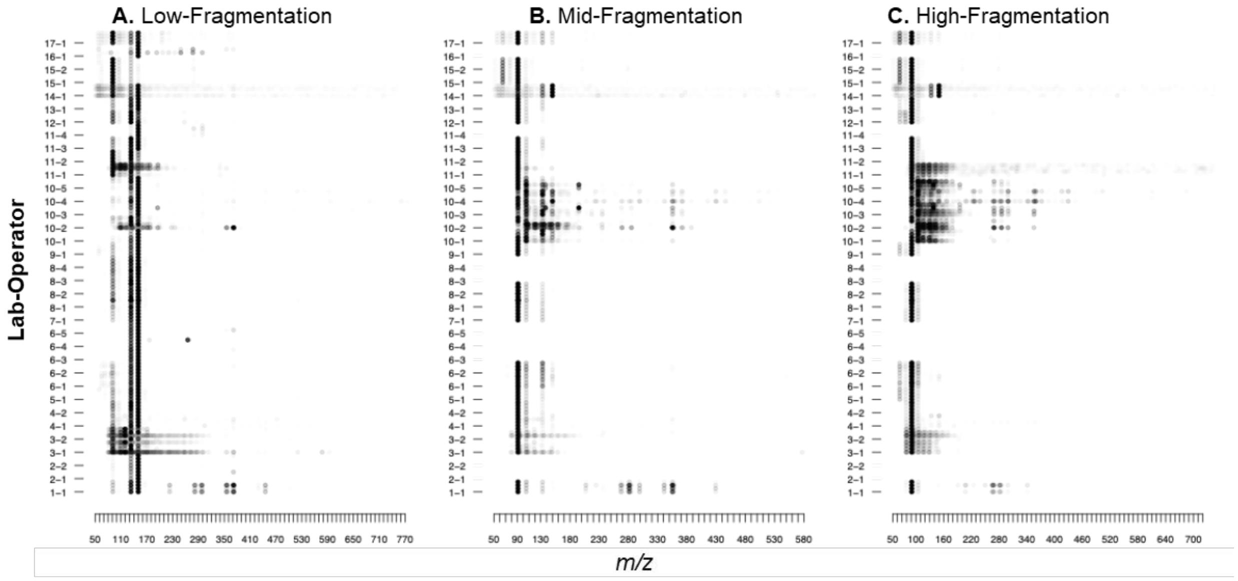

Figure S11. Example heat maps using aggregated is-CID mass spectral measurements of phentermine at (A.) low-fragmentation energy, (B.) mid-fragmentation energy, and (C.) high-fragmentation energy. Each operator completed four measurement sessions, resulting in four aggregated spectra per operator. Note that not all operators provided measurements at the mid- and high-fragmentation levels.

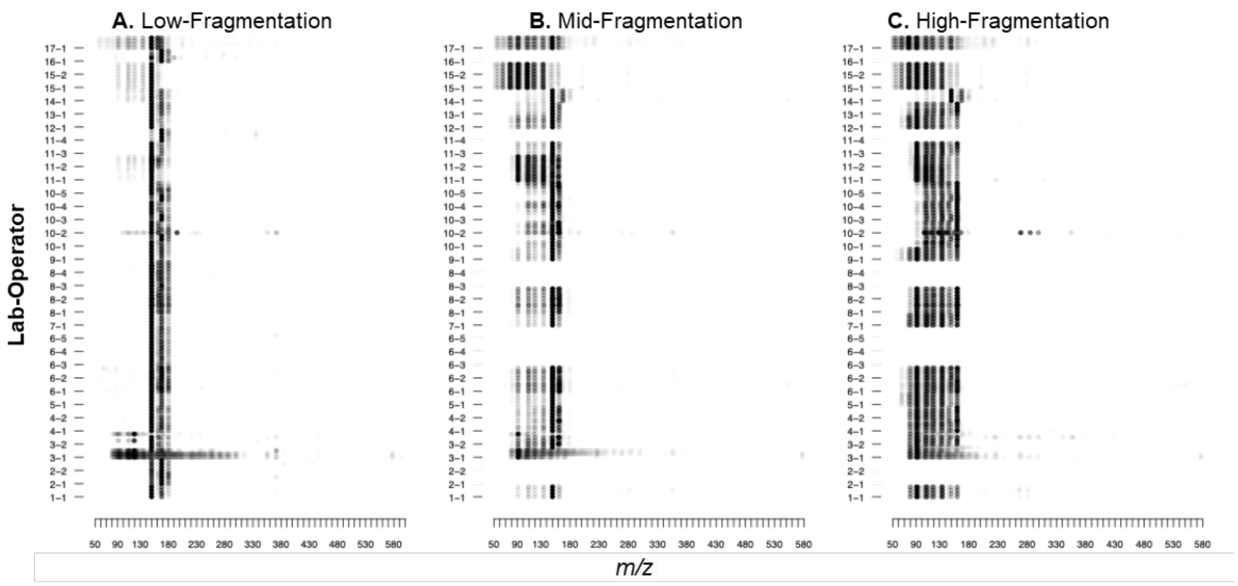

Figure S12. Example heat maps using aggregated is-CID mass spectral measurements of phenylephrine at (A.) low-fragmentation energy, (B.) mid-fragmentation energy, and (C.) high-fragmentation energy. Each operator completed four measurement sessions, resulting in four aggregated spectra per operator. Note that not all operators provided measurements at the mid- and high-fragmentation levels.

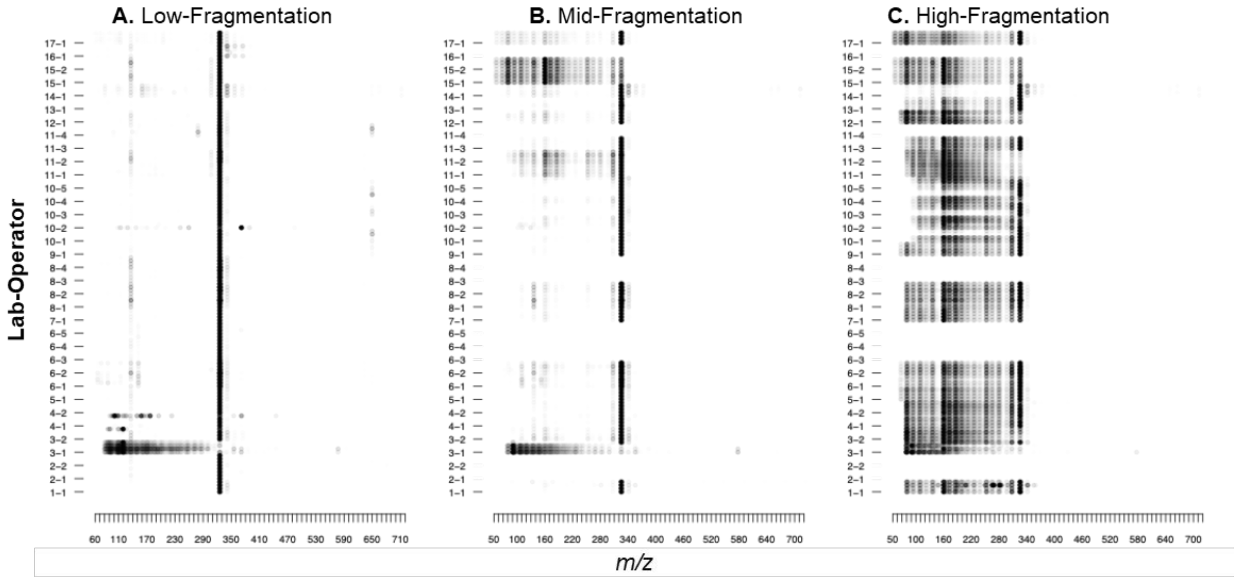

Figure S13. Example heat maps using aggregated is-CID mass spectral measurements of quinine at (A.) low-fragmentation energy, (B.) mid-fragmentation energy, and (C.) high-fragmentation energy. Each operator completed four measurement sessions, resulting in four aggregated spectra per operator. Note that not all operators provided measurements at the mid- and high-fragmentation levels.

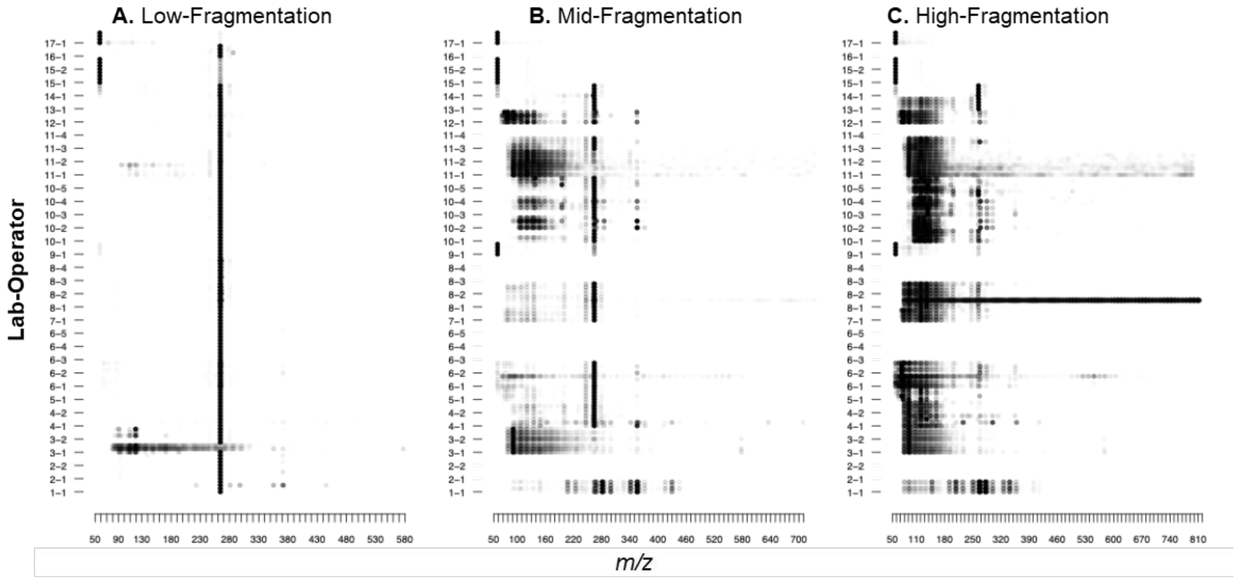

Figure S14. Example heat maps using aggregated is-CID mass spectral measurements of tramadol at (A.) low-fragmentation energy, (B.) mid-fragmentation energy, and (C.) high-fragmentation energy. Each operator completed four measurement sessions, resulting in four aggregated spectra per operator. Note that not all operators provided measurements at the mid- and high-fragmentation levels.

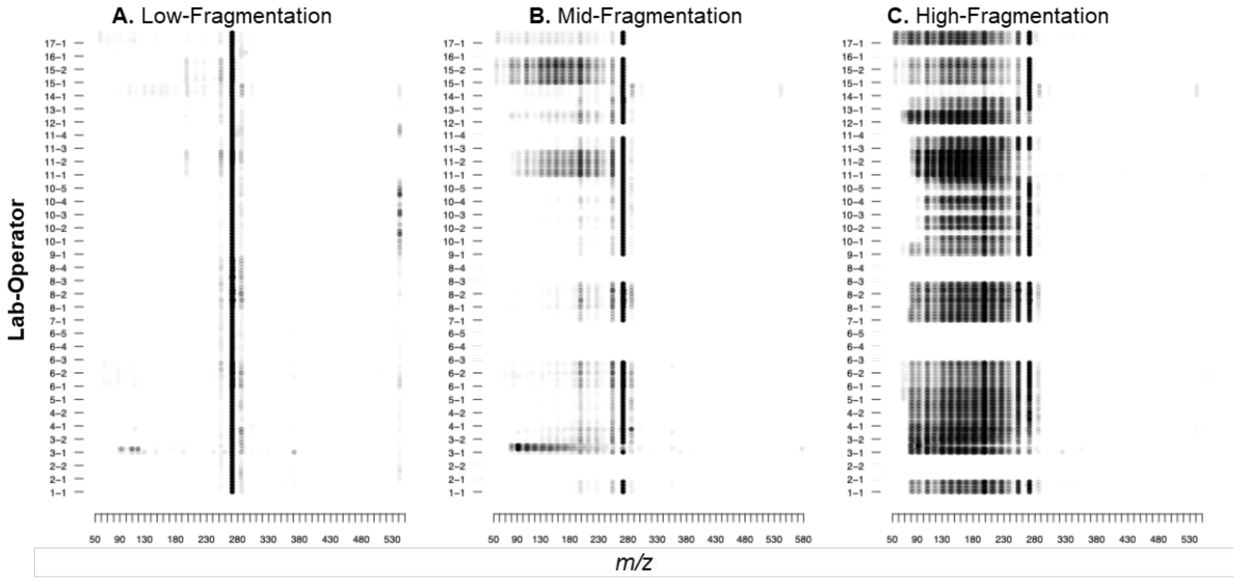

Figure S15. Example heat maps using aggregated is-CID mass spectral measurements of trenbolone at (A.) low-fragmentation energy, (B.) mid-fragmentation energy, and (C.) high-fragmentation energy. Each operator completed four measurement sessions, resulting in four aggregated spectra per operator. Note that not all operators provided measurements at the mid- and high-fragmentation levels.

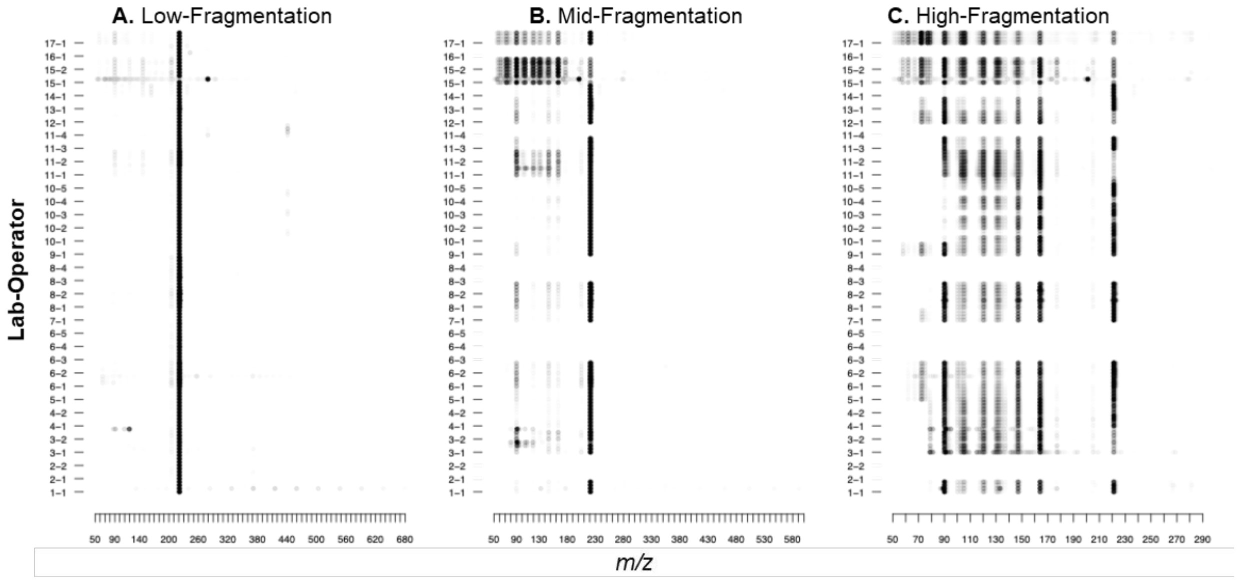

Figure S16. Example heat maps using aggregated is-CID mass spectral measurements of xylazine at (A.) low-fragmentation energy, (B.) mid-fragmentation energy, and (C.) high-fragmentation energy. Each operator completed four measurement sessions, resulting in four aggregated spectra per operator. Note that not all operators provided measurements at the mid- and high-fragmentation levels.

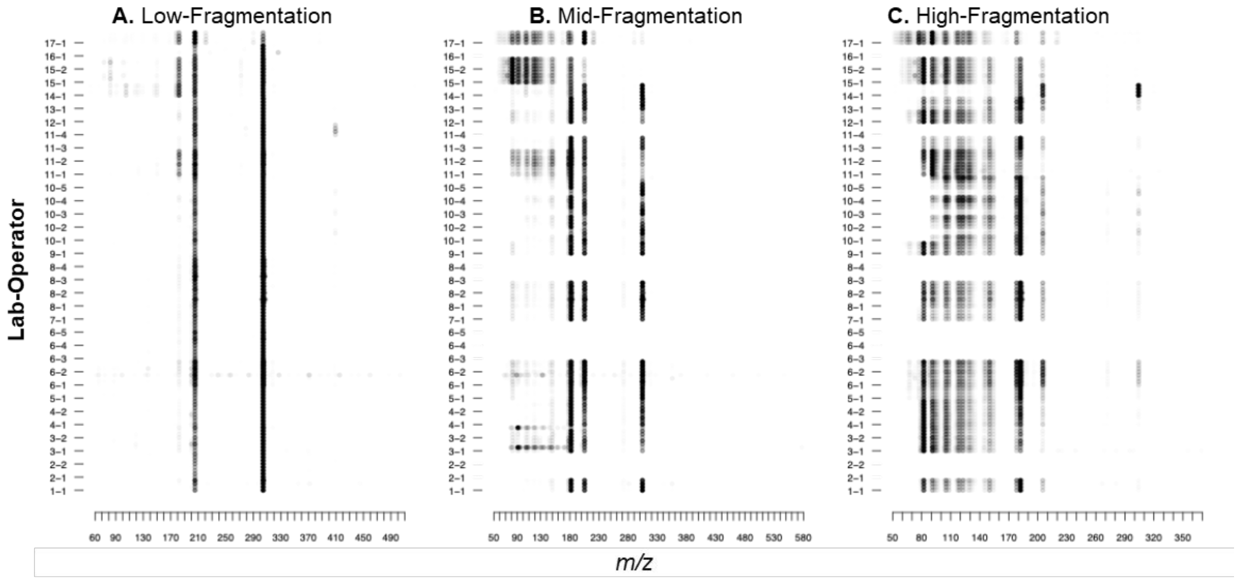

Figure S17. Example heat maps using aggregated is-CID mass spectral measurements of Mix 1 at (A.) low-fragmentation energy, (B.) mid-fragmentation energy, and (C.) high-fragmentation energy. Each operator completed four measurement sessions, resulting in four aggregated spectra per operator. Note that not all operators provided measurements at the mid- and high-fragmentation levels.

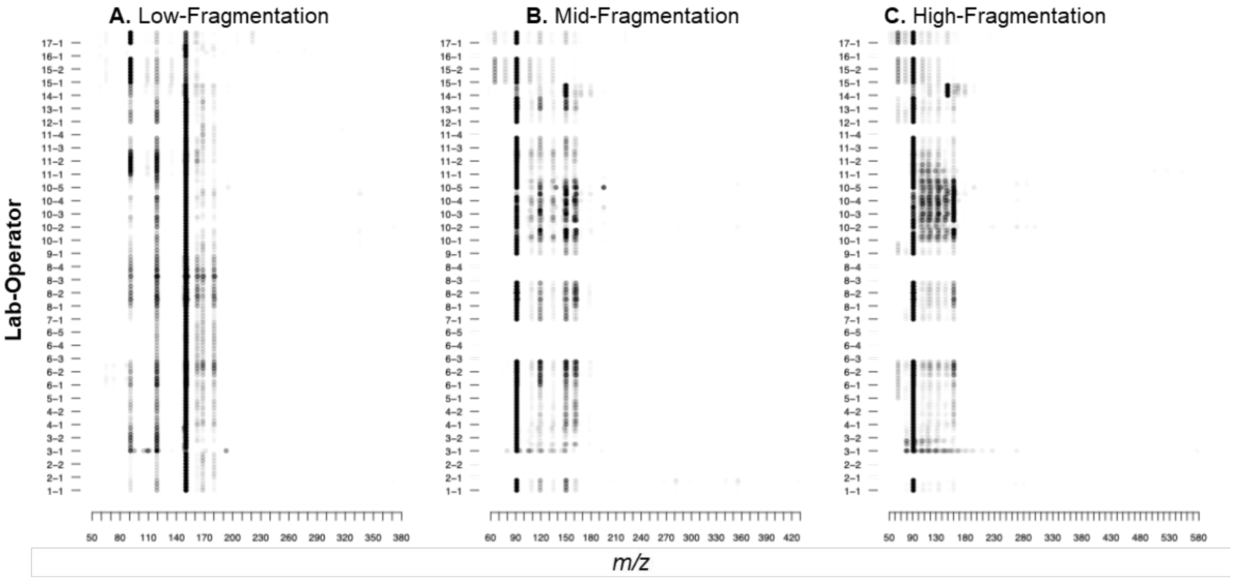

Figure S18. Example heat maps using aggregated is-CID mass spectral measurements of Mix 3 at (A.) low-fragmentation energy, (B.) mid-fragmentation energy, and (C.) high-fragmentation energy. Each operator completed four measurement sessions, resulting in four aggregated spectra per operator. Note that not all operators provided measurements at the mid- and high-fragmentation levels.

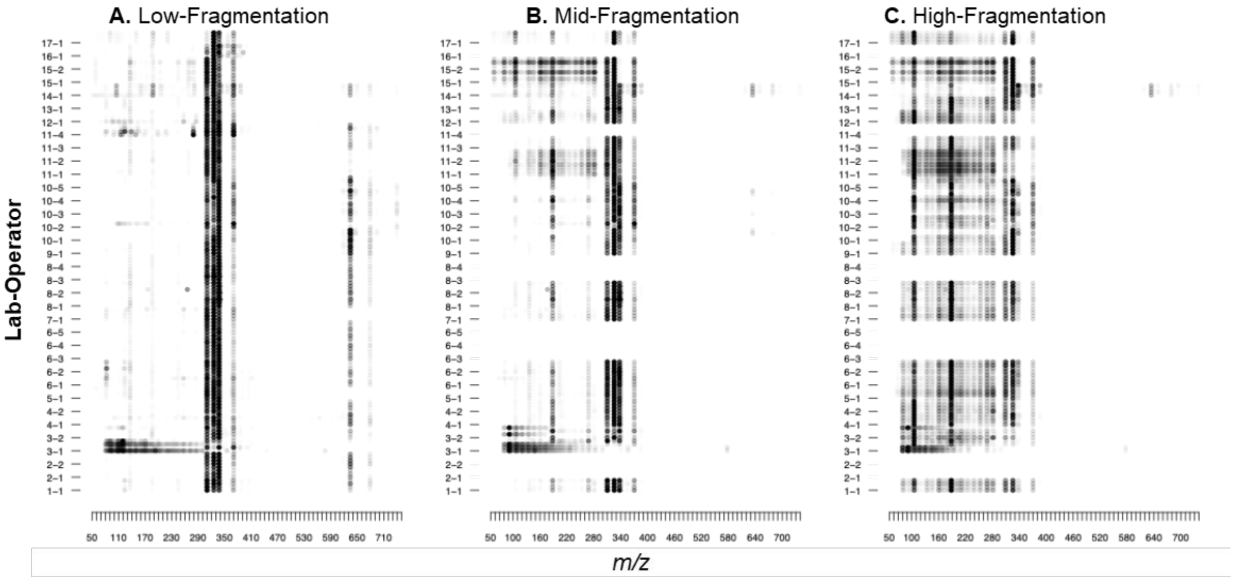

Figure S19. Example heat maps using aggregated is-CID mass spectral measurements of Mix 4 at (A.) low-fragmentation energy, (B.) mid-fragmentation energy, and (C.) high-fragmentation energy. Each operator completed four measurement sessions, resulting in four aggregated spectra per operator. Note that not all operators provided measurements at the mid- and high-fragmentation levels.

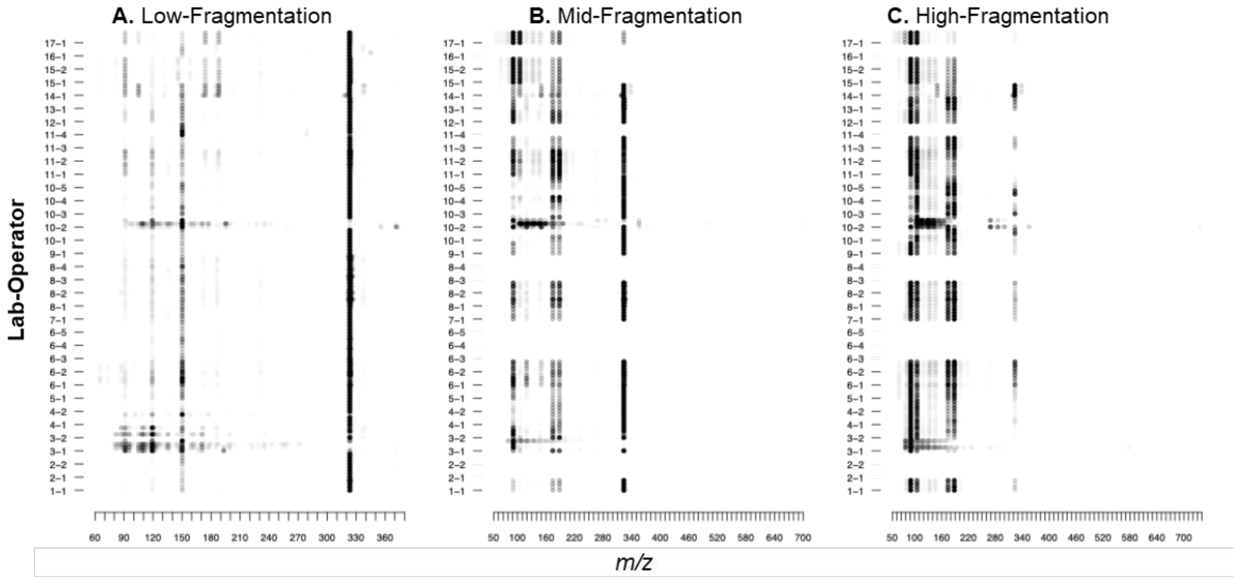

Figure S20. Example heat maps using aggregated is-CID mass spectral measurements of Mix 5 at (A.) low-fragmentation energy, (B.) mid-fragmentation energy, and (C.) high-fragmentation energy. Each operator completed four measurement sessions, resulting in four aggregated spectra per operator. Note that not all operators provided measurements at the mid- and high-fragmentation levels.

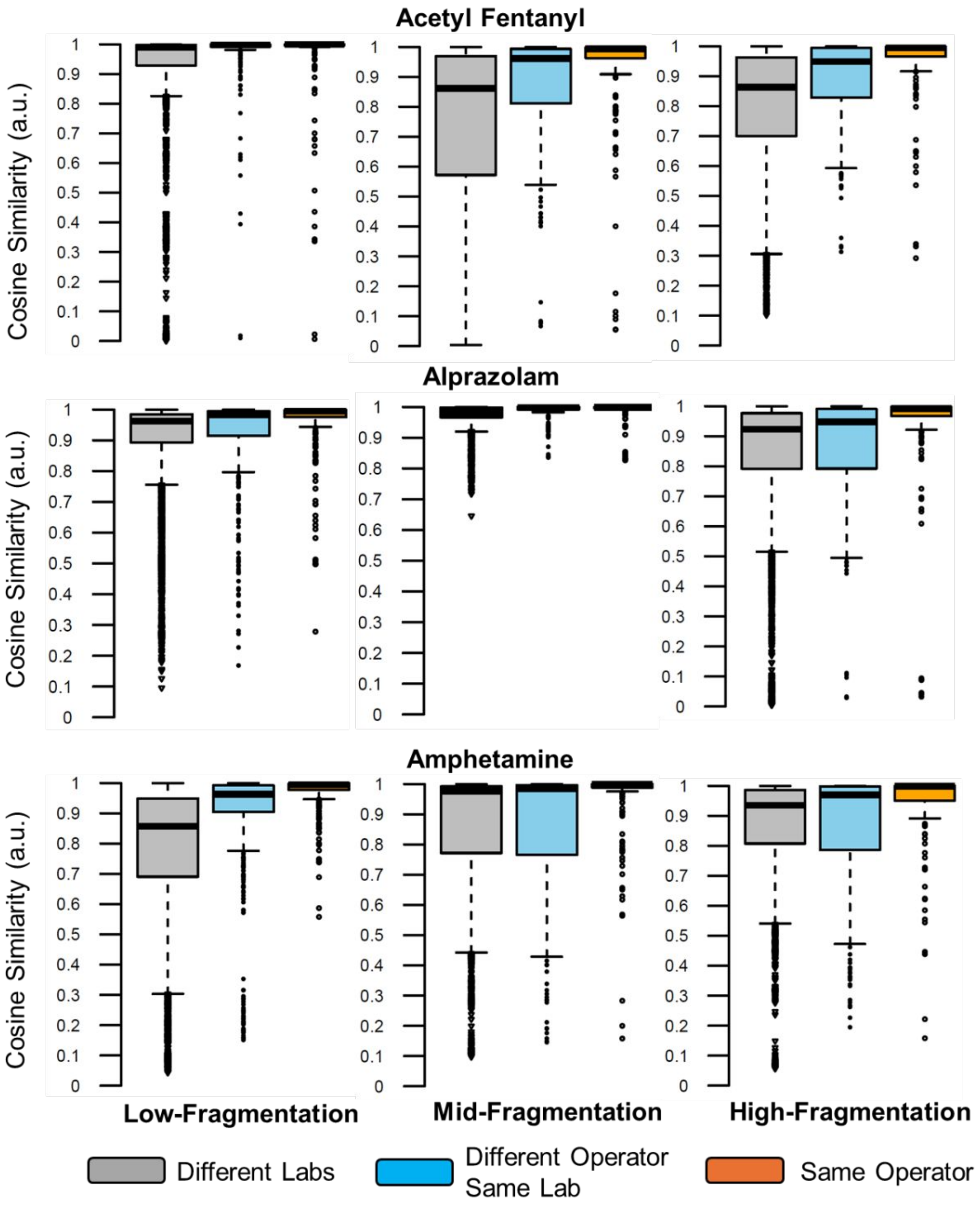

Figure S21. All computed similarity scores of spectra collected of the same sample visualized as a box-and-whisker plot, for acetyl fentanyl (top row), alprazolam (center row), and amphetamine (bottom row) collected at low-fragmentation *is*-CID (left column), mid-fragmentation *is*-CID (center column), and high-fragmentation *is*-CID (right column).

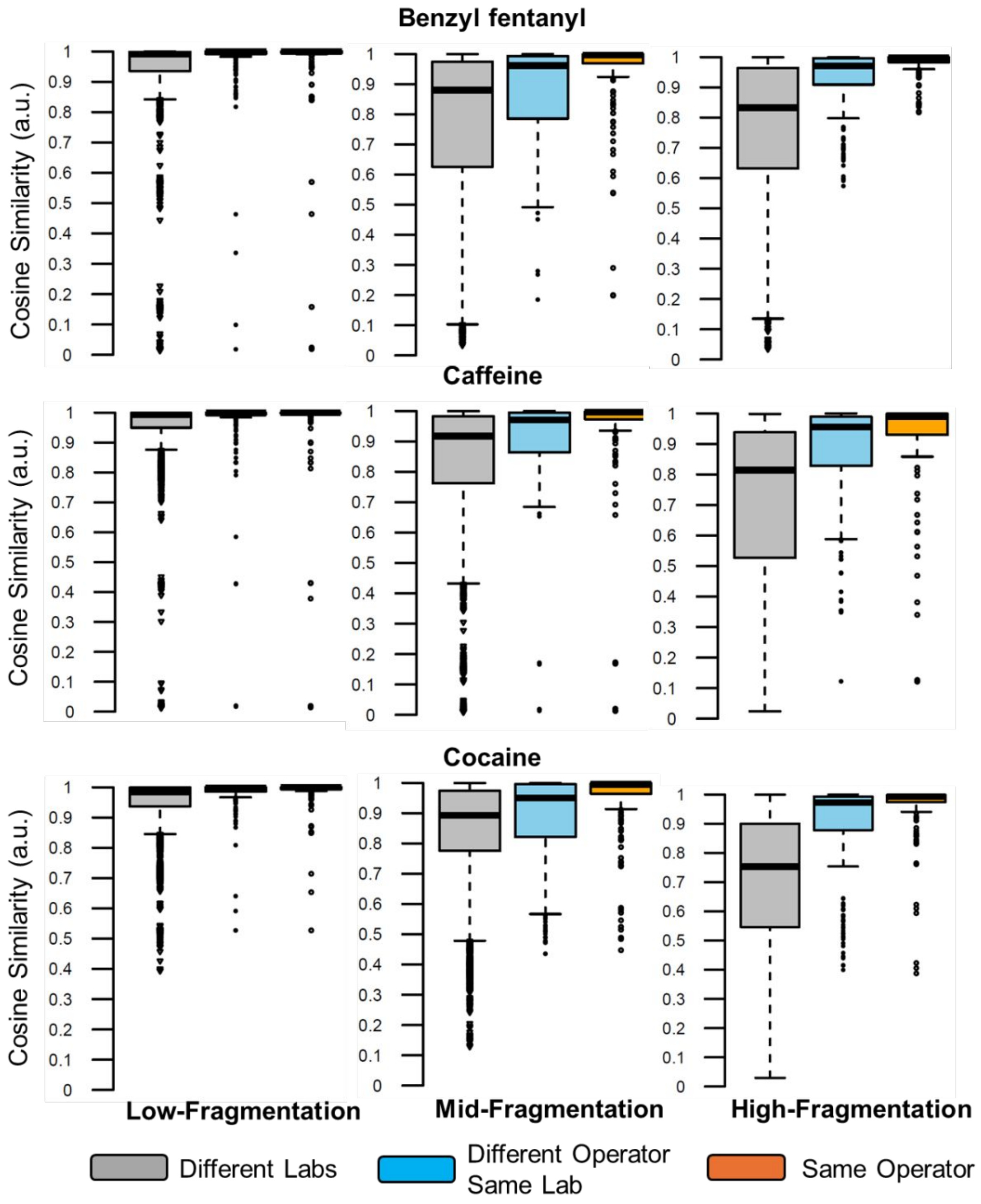

Figure S22. All computed similarity scores of spectra collected of the same sample visualized as a box-and-whisker plot, for benzyl fentanyl (top row), caffeine (center row), and cocaine (bottom row) collected at low-fragmentation *is*-CID (left column), mid-fragmentation *is*-CID (center column), and high-fragmentation *is*-CID (right column).

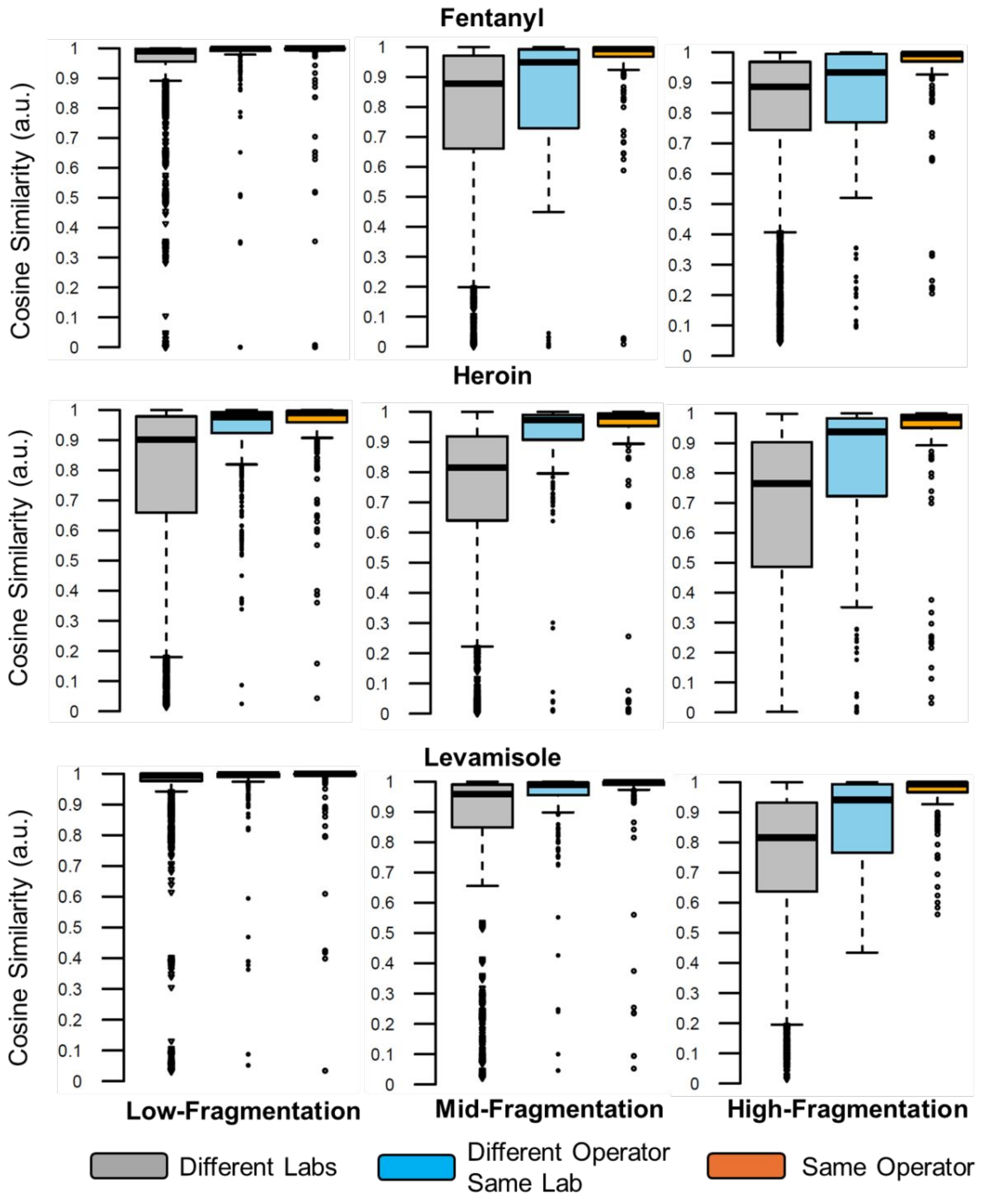

Figure S23. All computed similarity scores of spectra collected of the same sample visualized as a box-and-whisker plot, for fentanyl (top row), heroin (center row), and levamisole (bottom row) collected at low-fragmentation *is*-CID (left column), mid-fragmentation *is*-CID (center column), and high-fragmentation *is*-CID (right column).

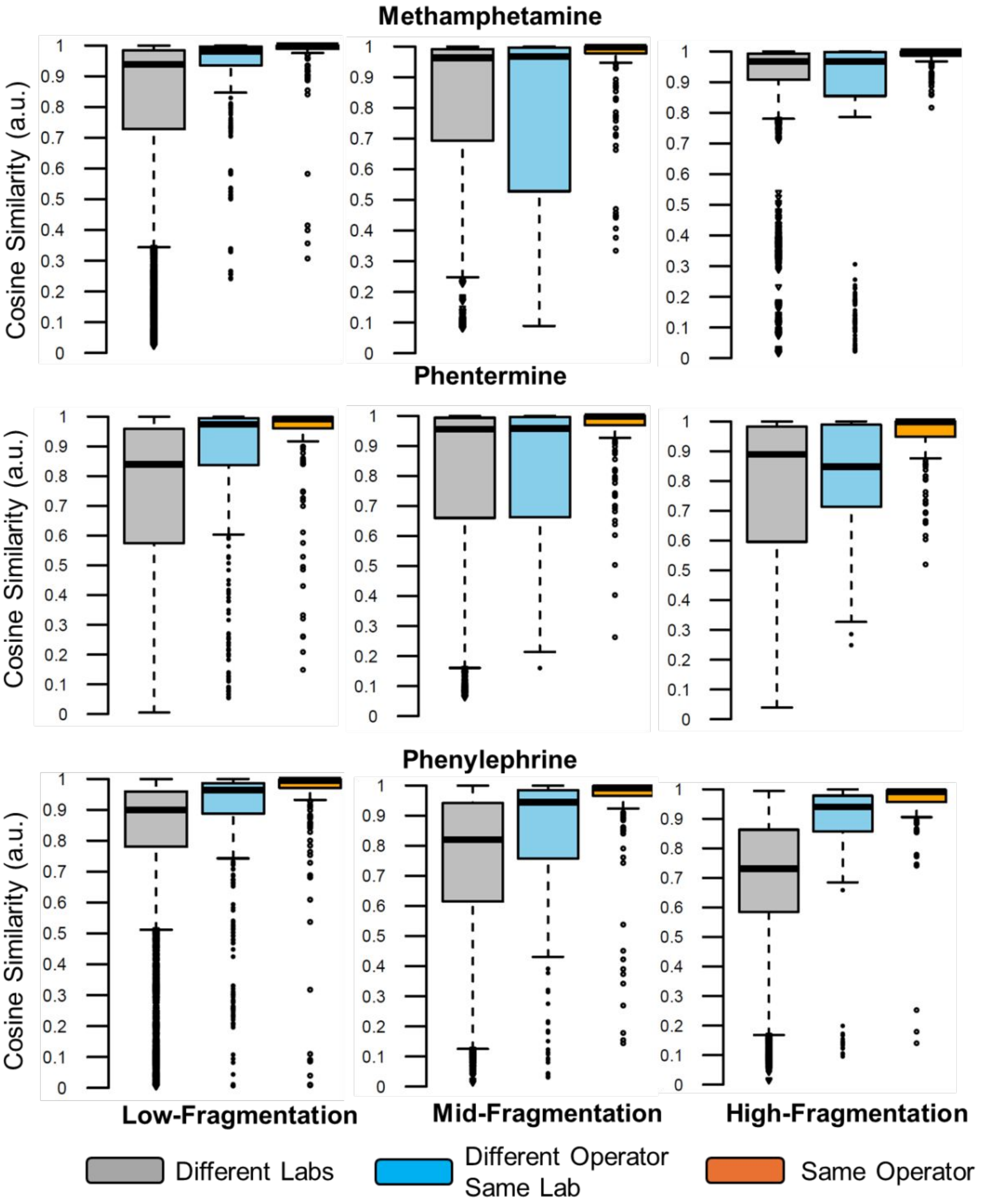

Figure S24. All computed similarity scores of spectra collected of the same sample visualized as a box-and-whisker plot, for methamphetamine (top row), phentermine (center row), and phenylephrine (bottom row) collected at low-fragmentation *is*-CID (left column), mid-fragmentation *is*-CID (center column), and high-fragmentation *is*-CID (right column).

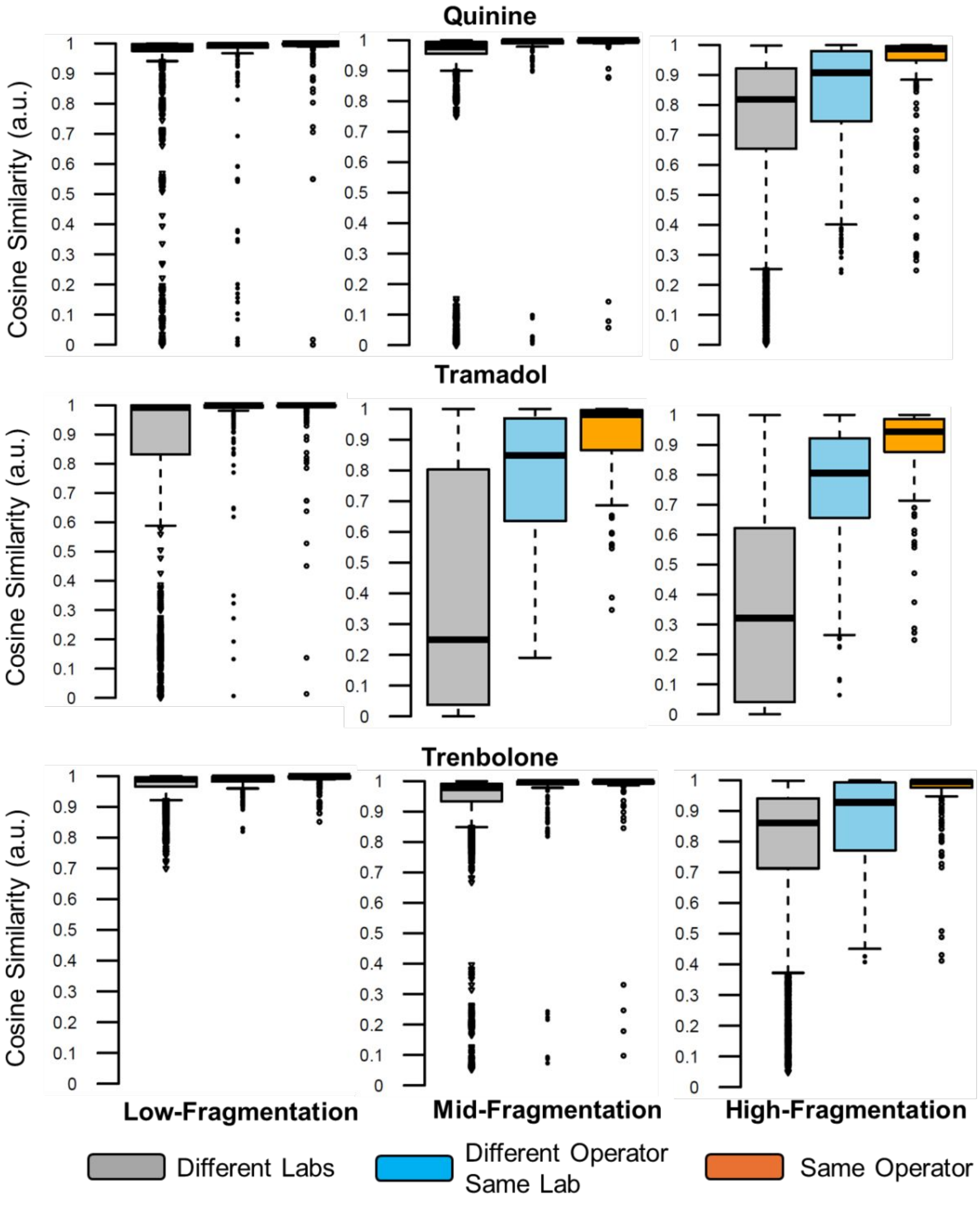

Figure S25. All computed similarity scores of spectra collected of the same sample visualized as a box-and-whisker plot, for quinine (top row), tramadol (center row), and trenbolone (bottom row) collected at low-fragmentation *is*-CID (left column), mid-fragmentation *is*-CID (center column), and high-fragmentation *is*-CID (right column).

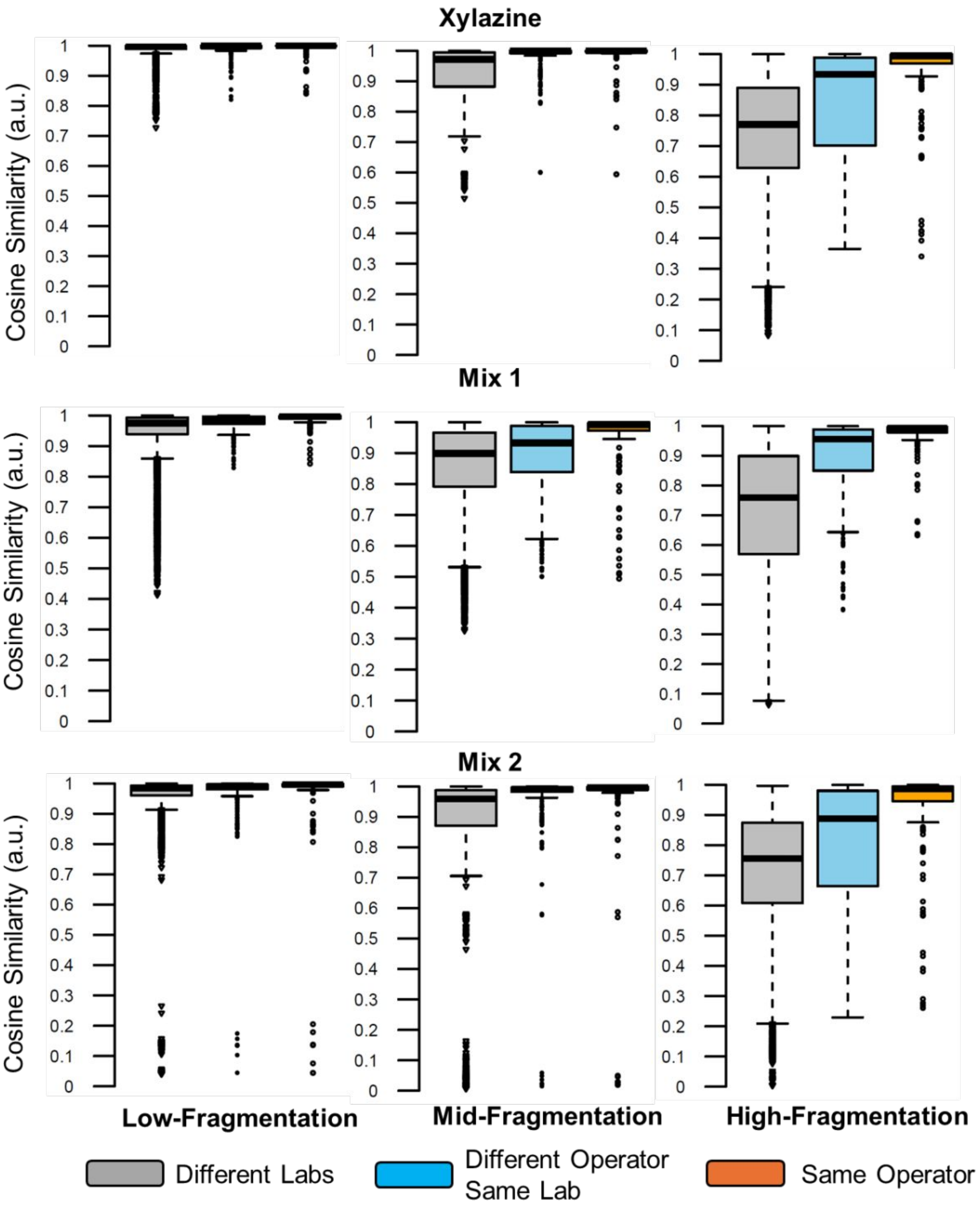

Figure S26. All computed similarity scores of spectra collected of the same sample visualized as a box-and-whisker plot, for xylazine (top row), Mix 1 (center row), and Mix 2 (bottom row) collected at low-fragmentation *is*-CID (left column), mid-fragmentation *is*-CID (center column), and high-fragmentation *is*-CID (right column).

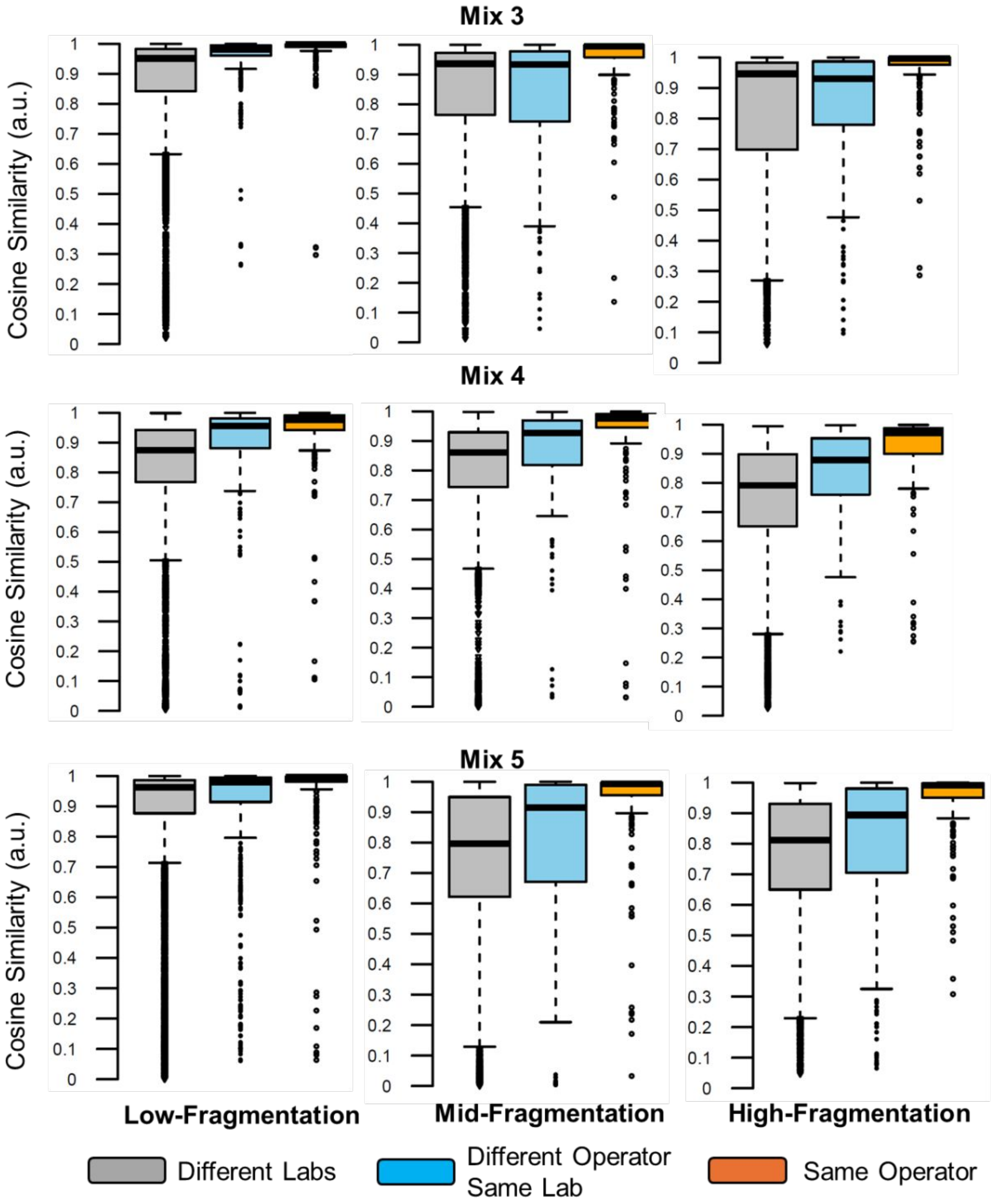

Figure S27. All computed similarity scores of spectra collected of the same sample visualized as a box-and-whisker plot, for Mix 3 (top row), Mix 4 (center row), and Mix 5 (bottom row) collected at low-fragmentation *is*-CID (left column), mid-fragmentation *is*-CID (center column), and high-fragmentation *is*-CID (right column).

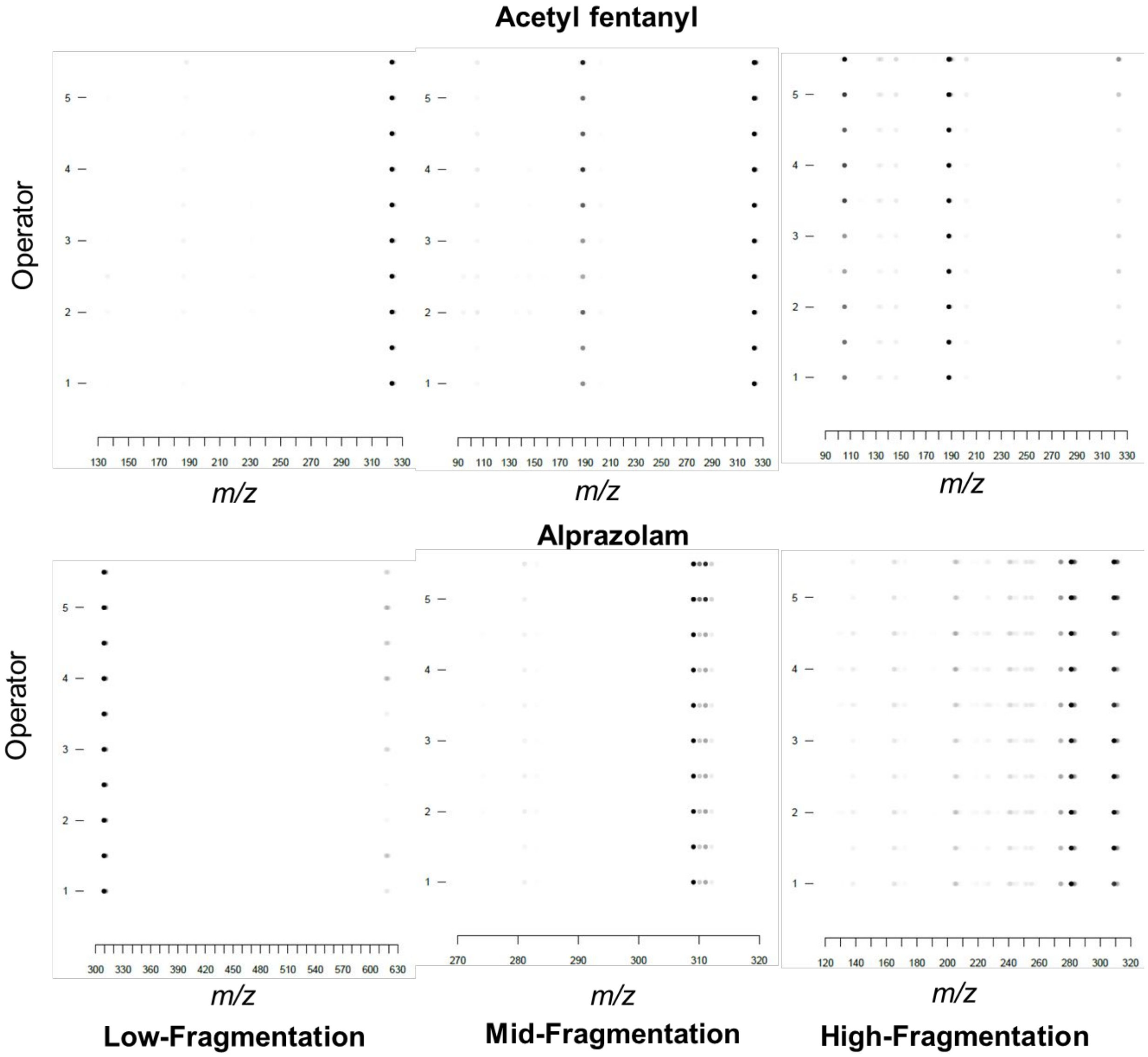

Figure S28. Heat maps of *is*-CID mass spectral measurements collected using the Uniform Method for acetyl fentanyl (top row) and alprazolam (bottom row) at low-fragmentation energy (left column), mid-fragmentation energy (center column), and high-fragmentation energy (right column). Each operator completed two measurement sessions.

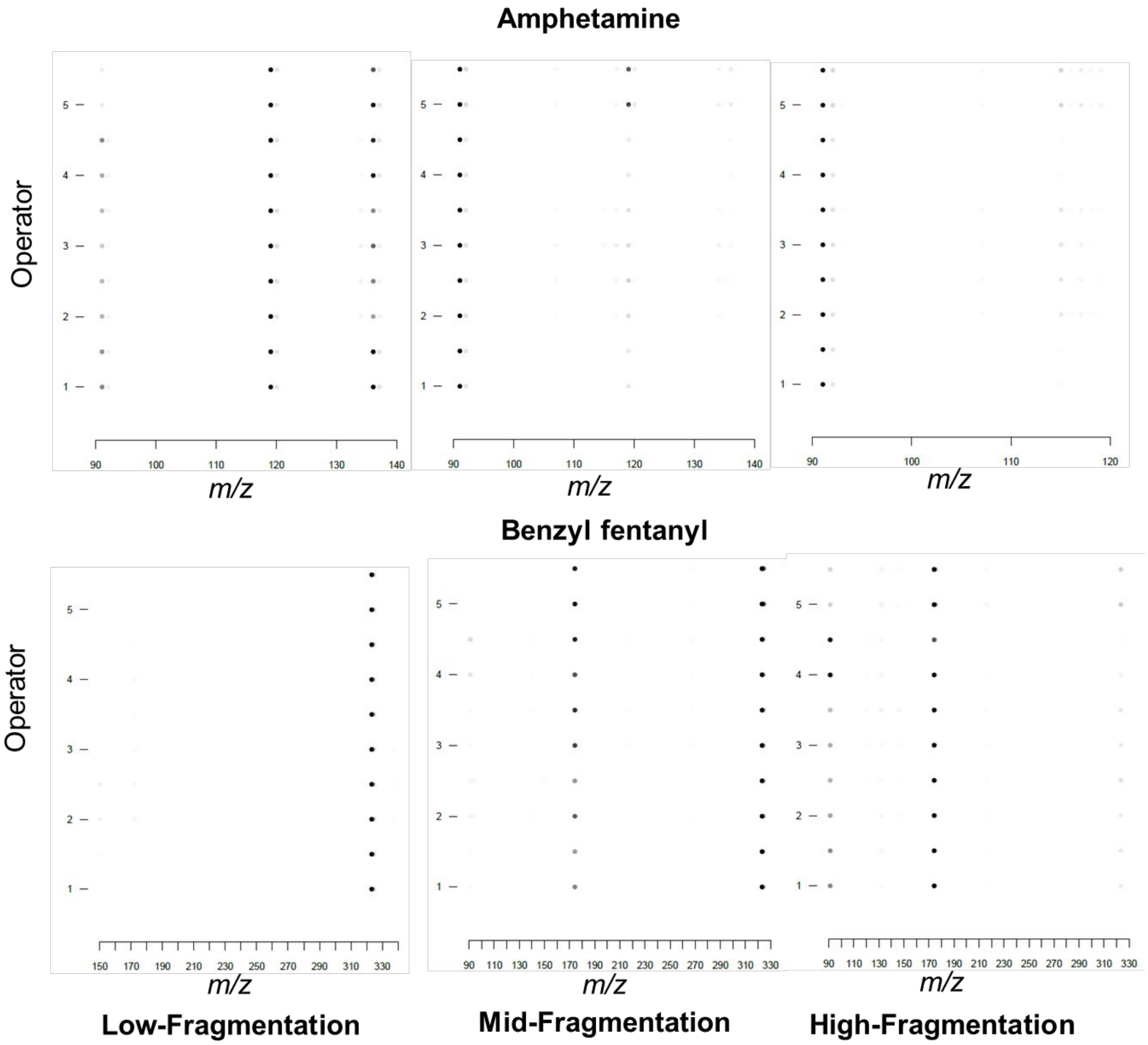

Figure S29. Heat maps of *is*-CID mass spectral measurements collected using the Uniform Method for amphetamine (top row) and benzyl fentanyl (bottom row) at low-fragmentation energy (left column), mid-fragmentation energy (center column), and high-fragmentation energy (right column). Each operator completed two measurement sessions.

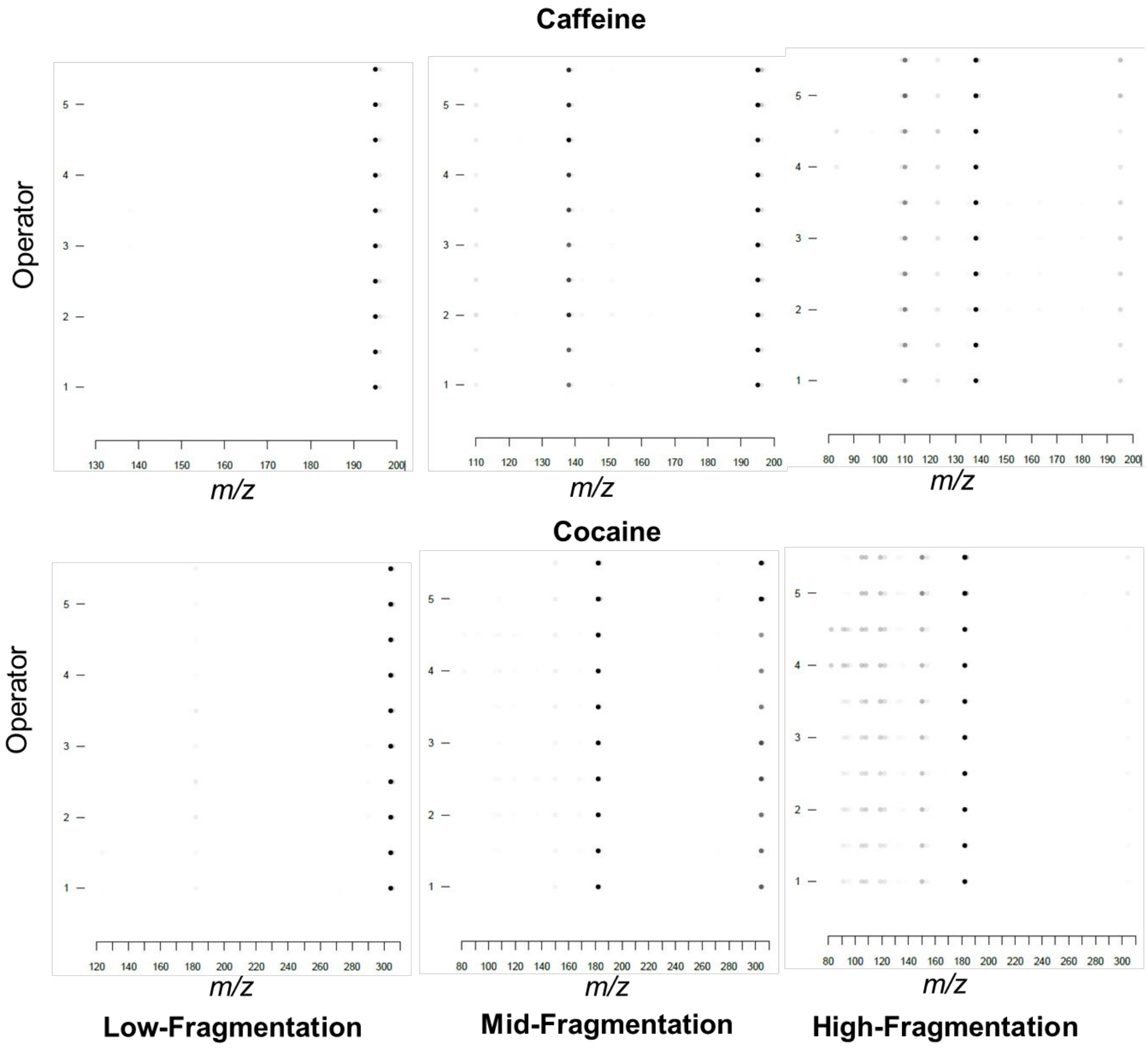

Figure S30. Heat maps of *is*-CID mass spectral measurements collected using the Uniform Method for caffeine (top row) and cocaine (bottom row) at low-fragmentation energy (left column), mid-fragmentation energy (center column), and high-fragmentation energy (right column). Each operator completed two measurement sessions.

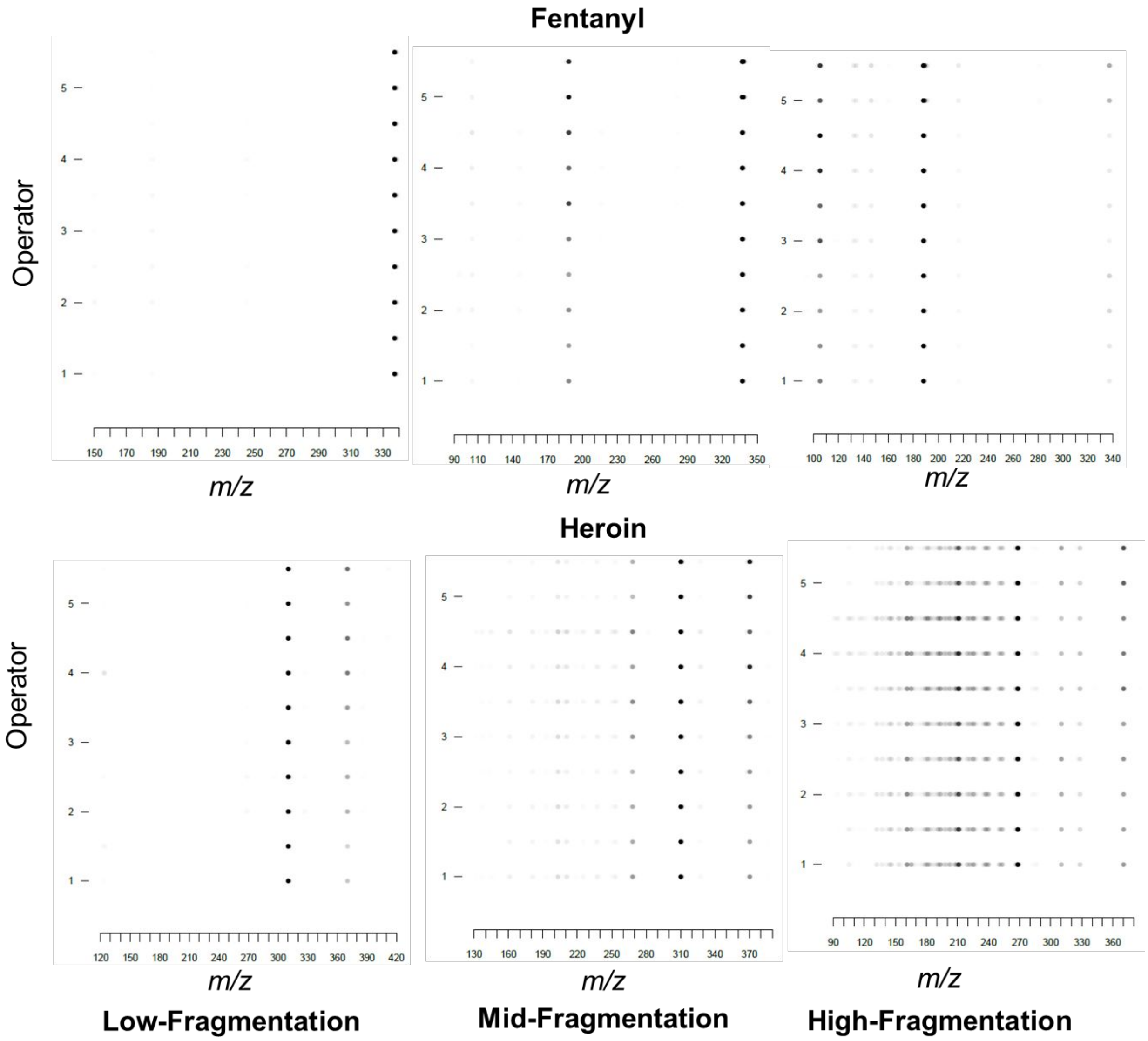

Figure S31. Heat maps of *is*-CID mass spectral measurements collected using the Uniform Method for fentanyl (top row) and heroin (bottom row) at low-fragmentation energy (left column), mid-fragmentation energy (center column), and high-fragmentation energy (right column). Each operator completed two measurement sessions.

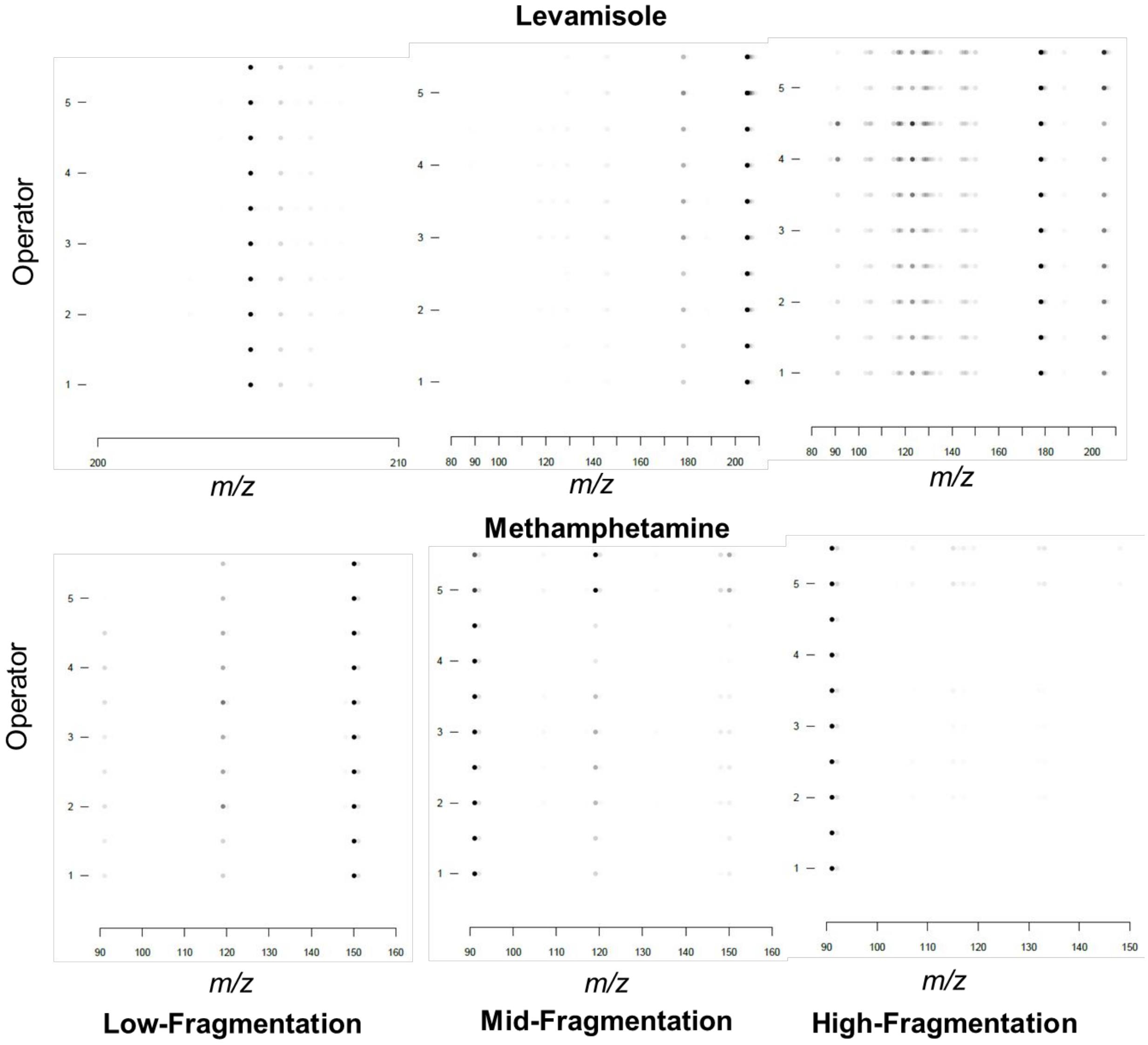

Figure S32. Heat maps of *is*-CID mass spectral measurements collected using the Uniform Method for levamisole (top row) and methamphetamine (bottom row) at low-fragmentation energy (left column), mid-fragmentation energy (center column), and high-fragmentation energy (right column). Each operator completed two measurement sessions.

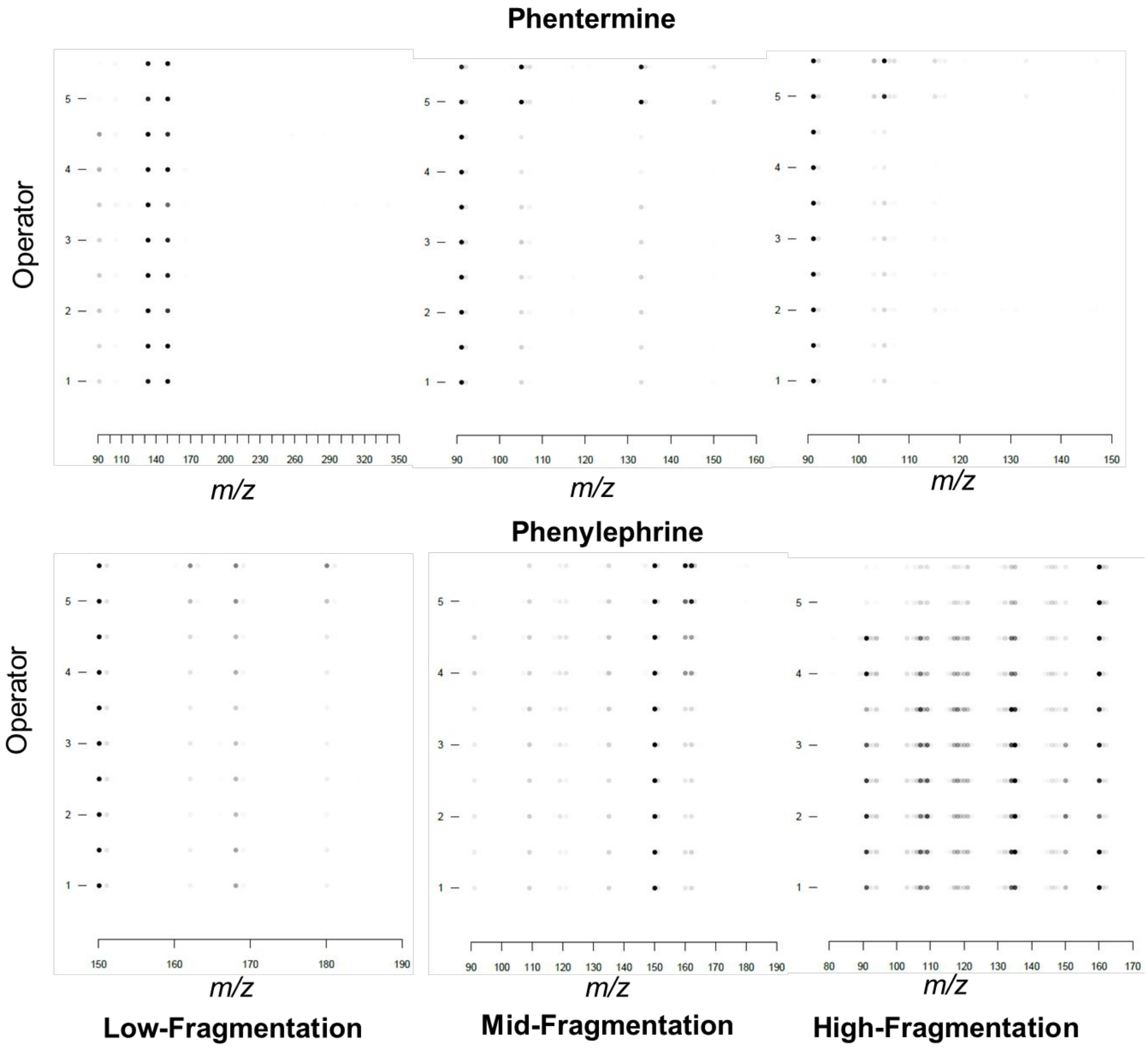

Figure S33. Heat maps of *is*-CID mass spectral measurements collected using the Uniform Method for phentermine (top row) and phenylephrine (bottom row) at low-fragmentation energy (left column), mid-fragmentation energy (center column), and high-fragmentation energy (right column). Each operator completed two measurement sessions.

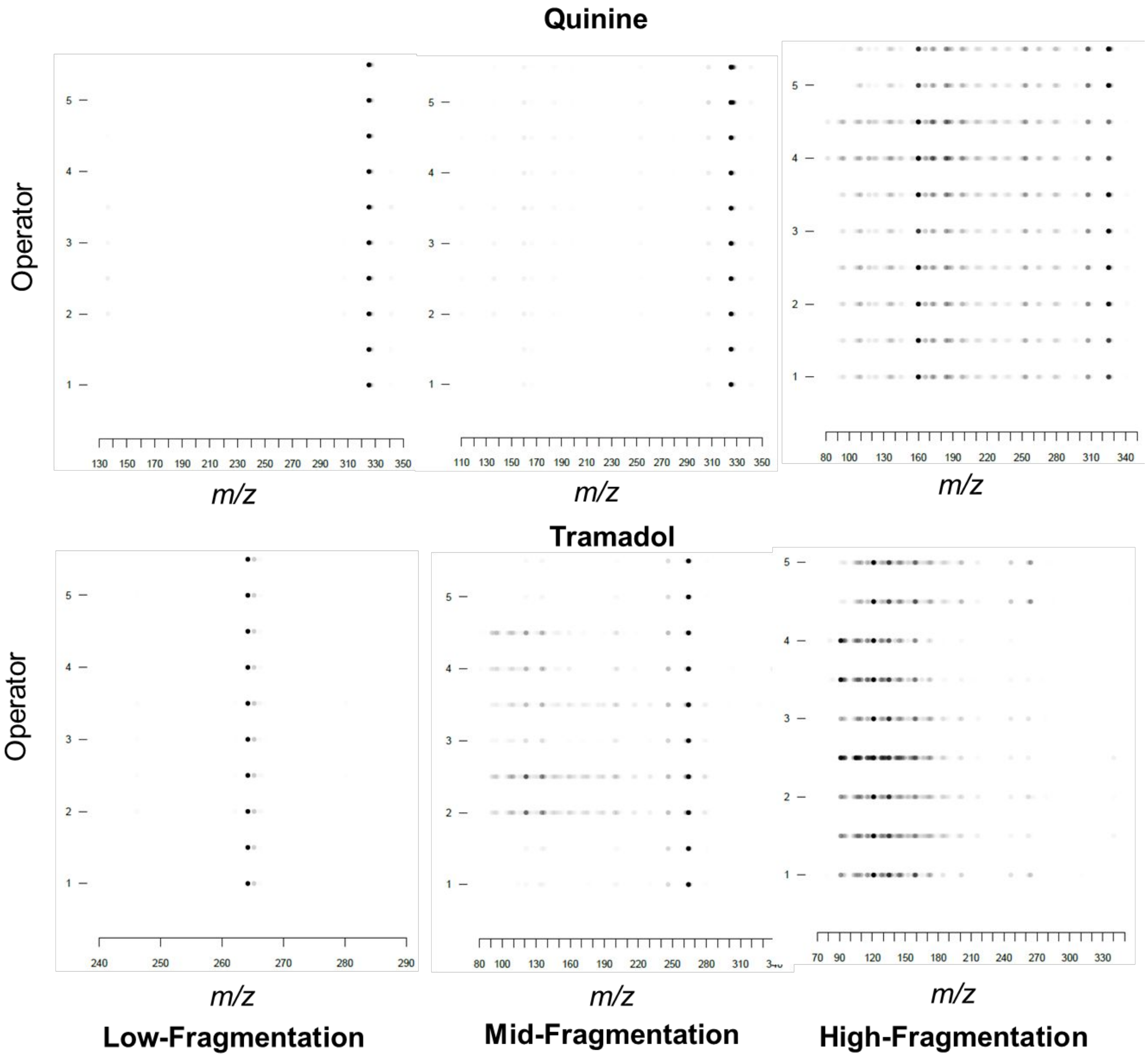

Figure S34. Heat maps of *is*-CID mass spectral measurements collected using the Uniform Method for quinine (top row) and tramadol (bottom row) at low-fragmentation energy (left column), mid-fragmentation energy (center column), and high-fragmentation energy (right column). Each operator completed two measurement sessions.

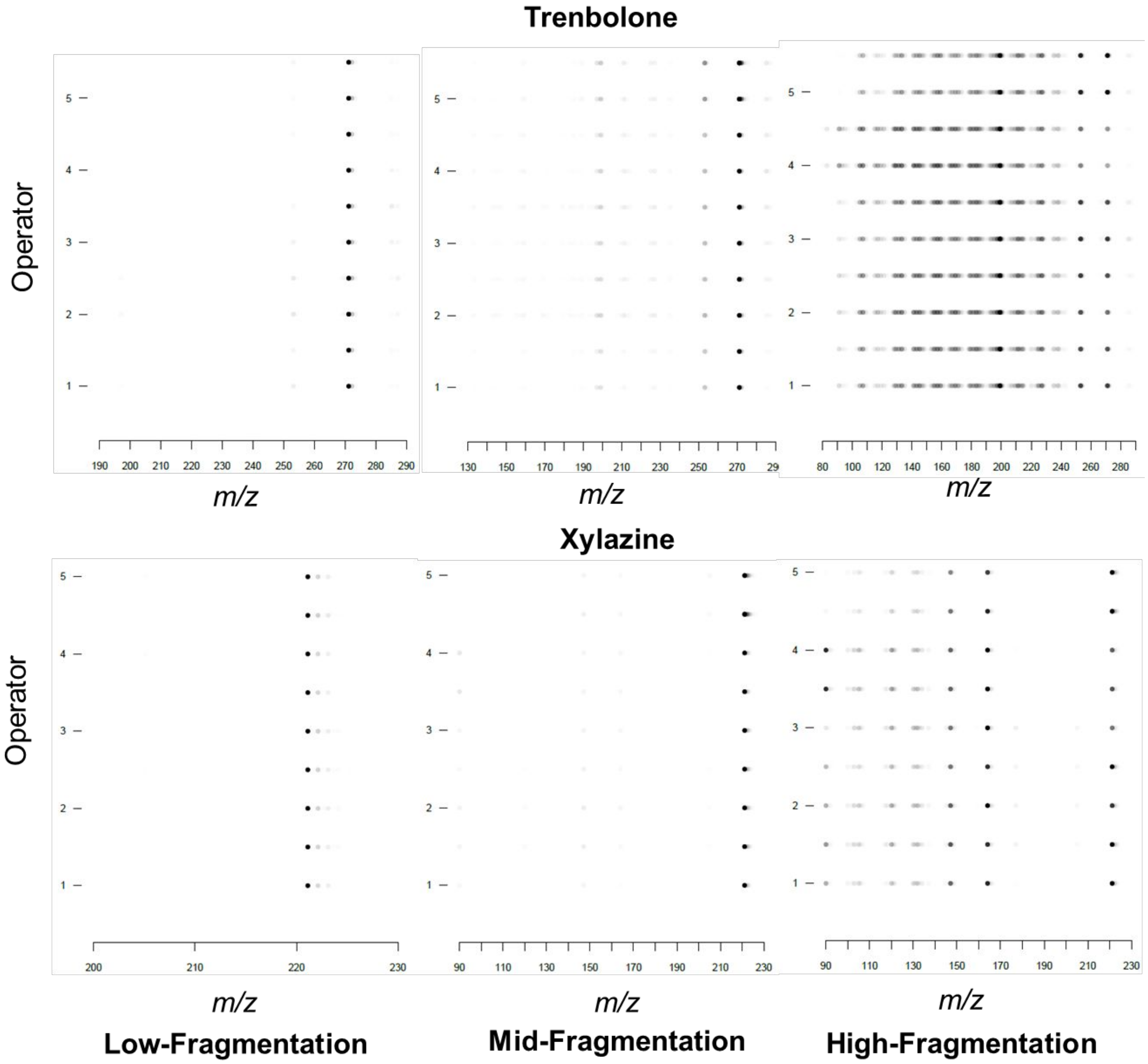

Figure S35. Heat maps of *is*-CID mass spectral measurements collected using the Uniform Method for trenbolone (top row) and xylazine (bottom row) at low-fragmentation energy (left column), mid-fragmentation energy (center column), and high-fragmentation energy (right column). Each operator completed two measurement sessions.

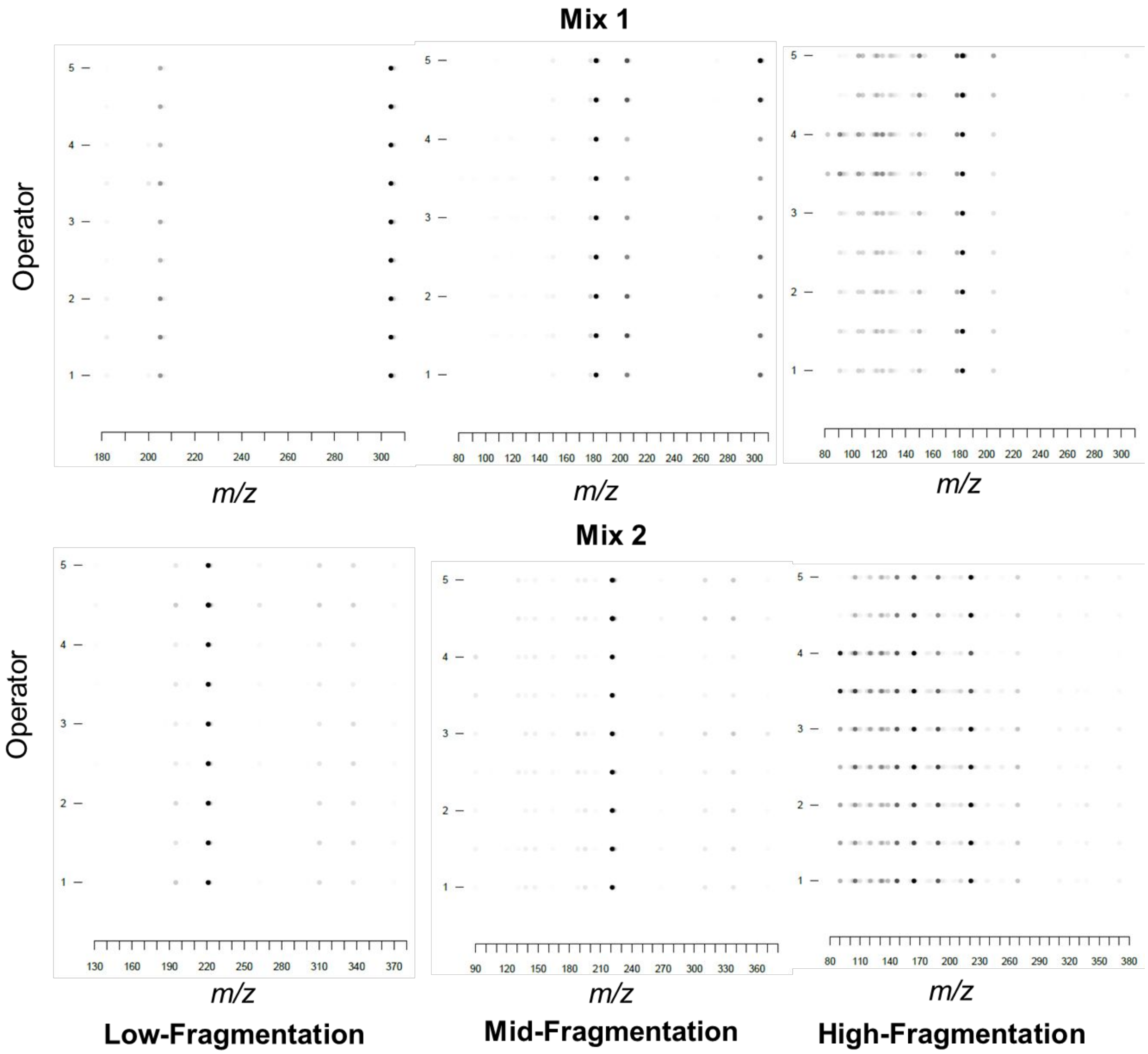

Figure S36. Heat maps of *is*-CID mass spectral measurements collected using the Uniform Method for Mix 1 (top row) and Mix 2 (bottom row) at low-fragmentation energy (left column), mid-fragmentation energy (center column), and high-fragmentation energy (right column). Each operator completed two measurement sessions.

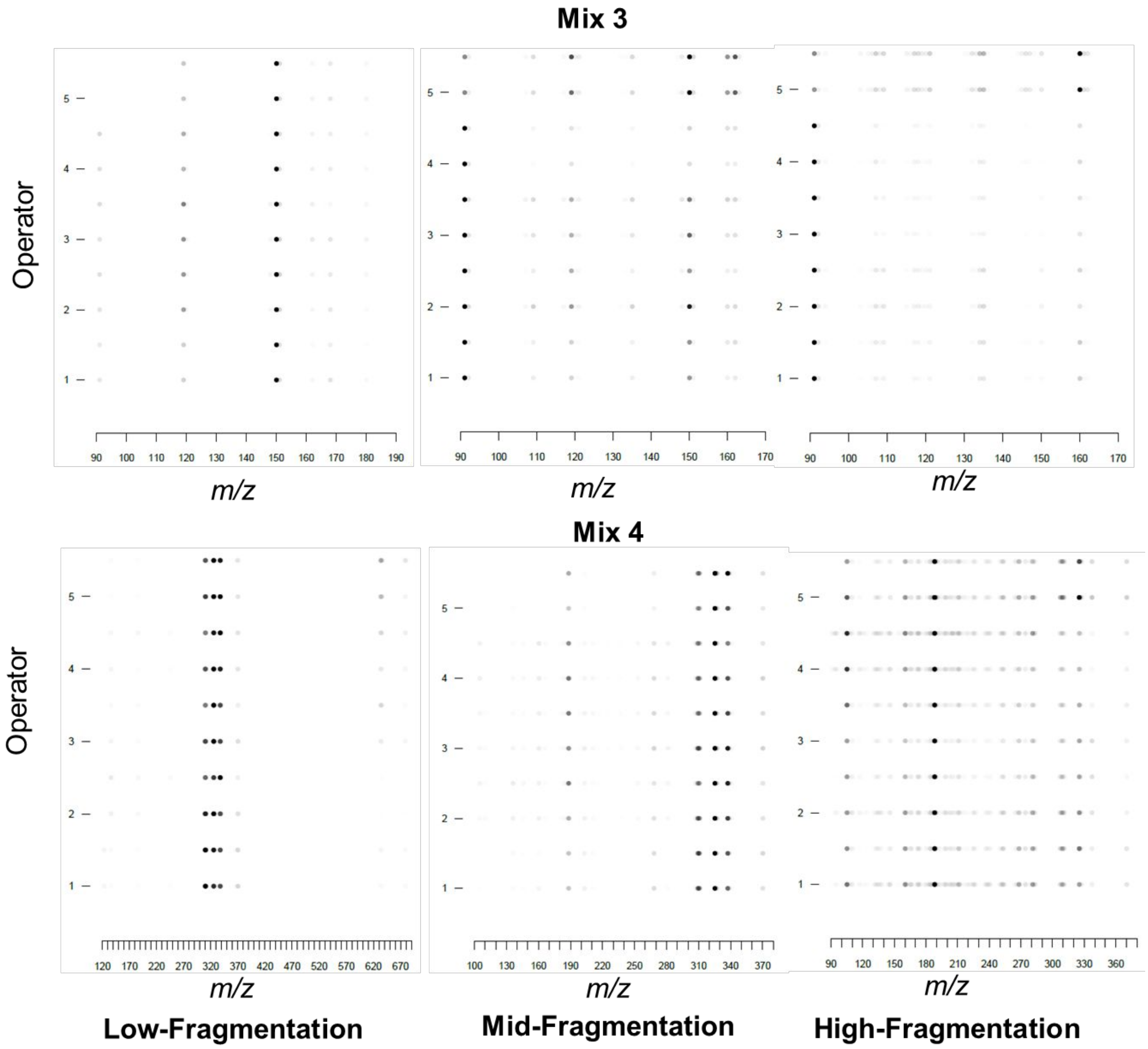

Figure S37. Heat maps of *is*-CID mass spectral measurements collected using the Uniform Method for Mix 3 (top row) and Mix 4 (bottom row) at low-fragmentation energy (left column), mid-fragmentation energy (center column), and high-fragmentation energy (right column). Each operator completed two measurement sessions.

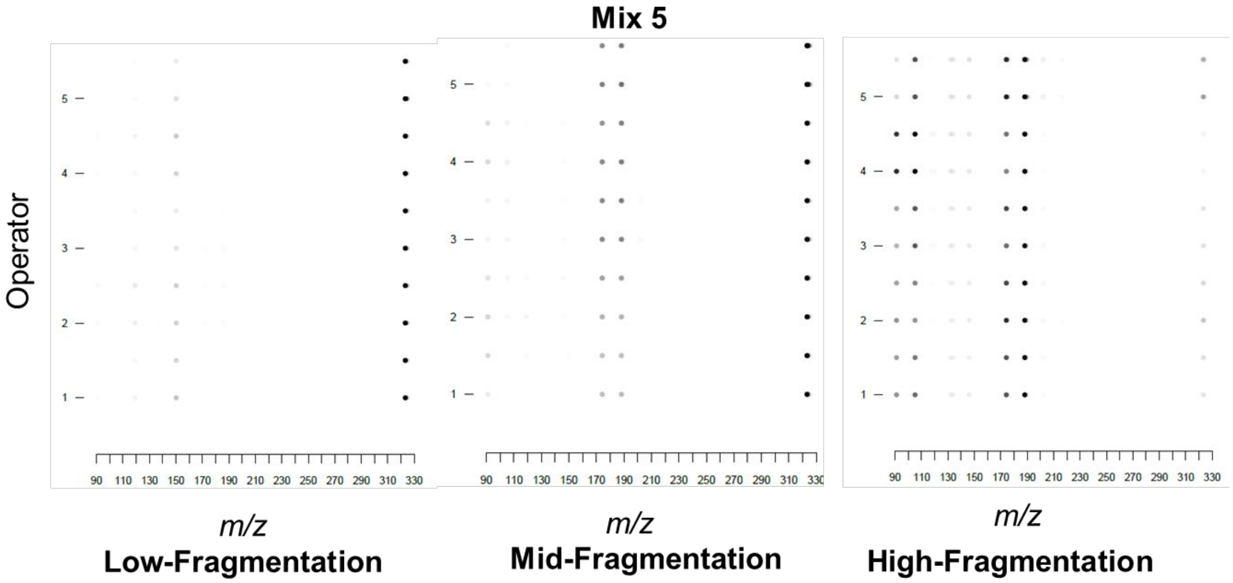

Figure S38. Heat maps of *is*-CID mass spectral measurements collected using the Uniform Method for Mix 5 at low-fragmentation energy (left column), mid-fragmentation energy (center column), and high-fragmentation energy (right column). Each operator completed two measurement sessions.
